# Novel flight style and light wings boost flight performance of tiny beetles

**DOI:** 10.1038/s41586-021-04303-7

**Published:** 2022-01-19

**Authors:** Sergey E. Farisenkov, Dmitry Kolomenskiy, Pyotr N. Petrov, Thomas Engels, Nadezhda A. Lapina, Fritz-Olaf Lehmann, Ryo Onishi, Hao Liu, Alexey A. Polilov

**Affiliations:** 1 Department of Entomology, Faculty of Biology, Lomonosov Moscow State University, Moscow, Russia; 2 Global Scientific Information and Computing Center, Tokyo Institute of Technology, Tokyo, Japan; 3 Skoltech Center for Design, Manufacturing and Materials, Skolkovo Institute of Science and Technology, Moscow, Russia; 4 Department of Animal Physiology, Institute of Biological Sciences, University of Rostock, Rostock, Germany; 5 Graduate School of Engineering, Chiba University, Chiba, Japan; 6 Joint Russian-Vietnamese Tropical Research and Technological Center, Southern Branch, Ho Chi Minh City, Vietnam

**Keywords:** Biophysics, Evolution, Entomology, Mechanical engineering, Biological physics

## Abstract

Flight speed is positively correlated with body size in animals^1^. However, miniature featherwing beetles can fly at speeds and accelerations of insects three times their size^2^. Here we show that this performance results from a reduced wing mass and a previously unknown type of wing-motion cycle. Our experiment combines three-dimensional reconstructions of morphology and kinematics in one of the smallest insects, the beetle *Paratuposa placentis* (body length 395 μm). The flapping bristled wings follow a pronounced figure-of-eight loop that consists of subperpendicular up and down strokes followed by claps at stroke reversals above and below the body. The elytra act as inertial brakes that prevent excessive body oscillation. Computational analyses suggest functional decomposition of the wingbeat cycle into two power half strokes, which produce a large upward force, and two down-dragging recovery half strokes. In contrast to heavier membranous wings, the motion of bristled wings of the same size requires little inertial power. Muscle mechanical power requirements thus remain positive throughout the wingbeat cycle, making elastic energy storage obsolete. These adaptations help to explain how extremely small insects have preserved good aerial performance during miniaturization, one of the factors of their evolutionary success.

## Abstract (web summary)

Three-dimensional reconstructions of morphology and flight mechanics of the beetle *Paratuposa placentis* reveal adaptations that enable extremely small insects to fly at speeds similar to those of much larger insects.

## Main

Driven by curiosity about the smallest objects, scientific exploration of the microscopic world has facilitated the miniaturization of various industrial products. But miniaturization is not just a human-made artifice: success stories of miniaturization are abundant in the living world. For more than 300 million years, ecological pressures have forced insects to develop extremely small bodiesdown to 200 μm long^3^without losing their ability to fly. As the physical properties of flight depend on size, constraints that are insignificant at the macro scale become significant at the micro scale, and vice versa^4^. Compared with larger sizes, flight at small sizes is dominated by viscous air friction rather than inertial forces resulting from the acceleration of the surrounding air. This competition between friction and inertia is key for flight at all size scales and thus applies to all animals that move through air.

**Fig. 1:**
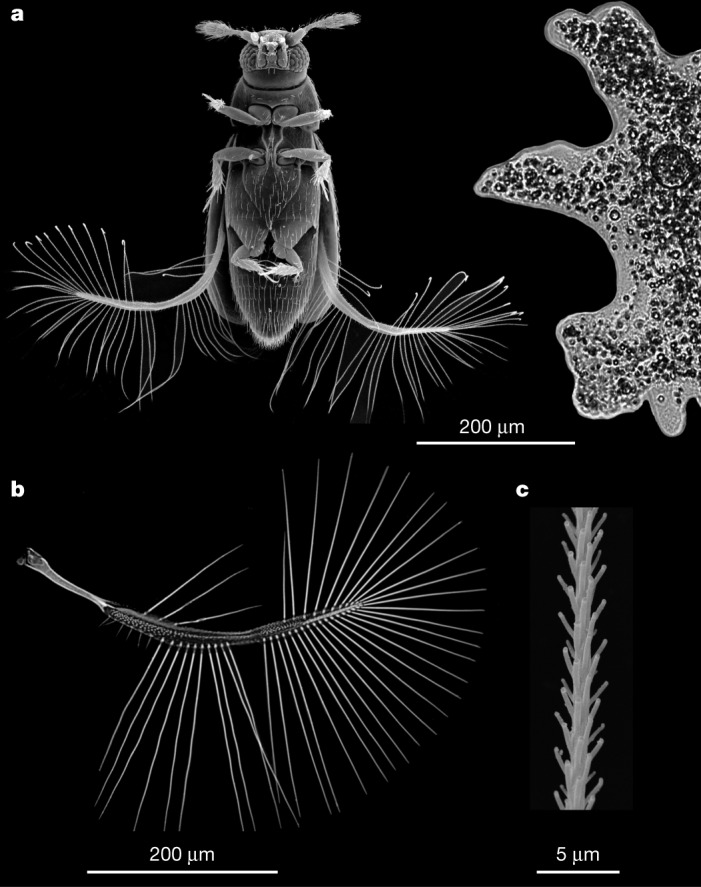
External morphology of *P. placentis*. **a**–**c**, Scanning electron microscopy images showing relative size of *P. placentis* (left) and *A. proteus* (right) (**a**), wing of *P. placentis* (**b**) and part of a seta (**c**). Every image was obtained from one randomly selected specimen; for detailed morphometry, see Supplementary Information.

Large insects generally fly faster than smaller ones^1^. Nevertheless, some of the smallest insects fly surprisingly well. For example, it was recently revealed that minute featherwing beetles (Coleoptera: Staphylinoidea: Ptiliidae) typically fly with similar speeds to their larger relatives (Staphylinidae), despite a threefold difference in body length^2^. Moreover, ptiliids can accelerate twice as fast as carrion beetles (Staphylinoidea: Silphidae), although the latter are an order of magnitude larger. As the size-specific flight-muscle volume is smaller in Ptiliidae than in larger beetles^5^, their excellent flight performance must result from the peculiar structure of their wings and flight style. Ptiliids have feather-like bristled wings—a condition known as ptiloptery (Fig. 1b)—instead of the membranous wings possessed by most insects. This visually striking modification of the flight apparatus evolved convergently in extremely small representatives of several insect orders. The functional benefits of ptiloptery, however, have remained largely unknown.

Although many studies have focused on the secrets of flight in minute insects^6,7^, most experimental data that elucidate wing motion and aerodynamics have been obtained from larger insect species^8–11^. Thus, unsteady aerodynamics of millimetre-size insects such as fruit flies^12,13^ and mosquitoes^14^ have received considerable attention in recent decades, whereas studies focusing on tiny insects remained scarce. Two-dimensional numerical studies on the aerodynamics of insect wings have previously shown that the flow past evenly spaced cylinder lattices reduces aerodynamic force production in bristled wings^15,16^. By contrast, experiments with mechanical comb-like models have suggested slightly larger lift-to-drag ratios during the clap-and-fling phase in bristled wings compared with membranous wings^17–19^, but did not cover the full wingbeat cycle. Meanwhile, using state-of-the-art high-speed videography, it has become clear that small insects use a wingbeat cycle that is different from that of the larger ones^10,11^, but, to our knowledge, the role of ptiloptery in this cycle has not been considered.

In this study, we analysed the flight of the miniature featherwing beetle *Paratuposa placentis*. We constructed a morphological model based on data gained from light, confocal and electron microscopy measurements, a kinematical model using synchronized high-speed videography, and a dynamical model using computational methods of solid and fluid mechanics. The combination of these methods offers a comprehensive view of how bristled wings work and explains why common sub-millimetre flying insects have bristled rather than membranous wings.

## Structural features of *P. placentis*

**Fig. 2:**
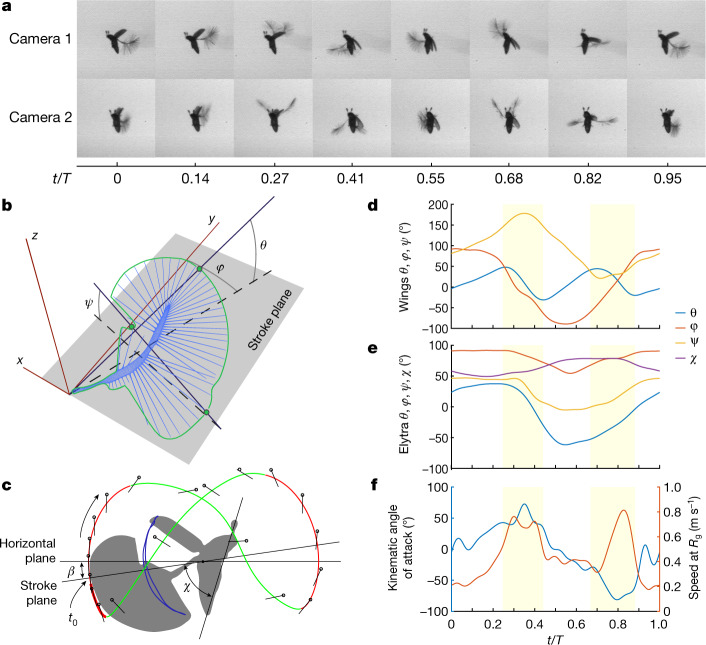
Kinematics of *P. placentis*. **a**, Frame sequence of a single stroke in two projections. **b**, Measurement scheme for Euler angles. **c**, Trajectory of the wing tip: recovery strokes (magenta line) and power strokes (green line) and measurement scheme for angle of body pitch (*χ*) and pitch of stroke plane to the horizon (*β*). **d**, Wing Euler angles as functions of dimensionless time *t*/*T*, *T* = 1/*f*: stroke deviation (*θ*), positional (*φ*) and pitch (*ψ*). **e**, Elytron Euler angles (*θ*, *φ* and *ψ*); body pitch angle (*χ*). **f**, AoA and wing speed at radius of gyration (*R*_g_) versus *t/T*.

*P. placentis* is one of the smallest non-parasitic insect species, with a body length of about 395 ± 21 μm (all measurements are given as mean ± s.d.). This size is similar to the size of some unicellular protists such as *Amoeba proteus* (Fig. 1a). The body mass of *P. placentis* is 2.43 ± 0.19 µg (Supplementary Information). The bristled wing consists of a petiole, a narrow wing blade and a fringe of setae (bristles) covered with secondary outgrowths (Fig. 1b, c). The wing length is 493 ± 18 μm and the setae occupy 95.1 ± 0.3% of the aerodynamically effective wing area (interior of the green contour in Fig. 2b).

## Wing kinematics

The wingbeat cycle of *P. placentis* consists of two power strokes, during which most of the total flight force is generated^20^, and two recovery strokes with wings clapping above and below the body (Fig. 2a, c, Supplementary Videos 1–6). Dorsal and ventral recovery strokes are unique to the Ptiliidae and replace the conventional clap-and-fling kinematics described in other insects, including miniature thrips^9^ and parasitoid wasps^8^. Despite the large stroke amplitude, the wings do not always clap tightly at the end of the ventral recovery stroke, depending on flight conditions (Supplementary Information). The setal fringes of the left and right wings may intersect during the fling phases of the recovery strokes. The morphological downstroke and upstroke are remarkably similar: the angle of attack (AoA) reaches 73° during the downstroke and 85° in magnitude during the upstroke (Fig. 2f). The cycle-averaged Reynolds number (Re) based on the mean speed of the radius of gyration is 9 and reaches 20 during power strokes when wing velocity is highest. The increased AoA during power strokes and the presence of recovery strokes are similar to the kinematics of swimming in miniature aquatic crustaceans (Supplementary Information), which move at similar flow regimes—for example, larvae of *Artemia* sp.^20^, with a Reynolds number of 10.

## Vertical force generation

**Fig. 3:**
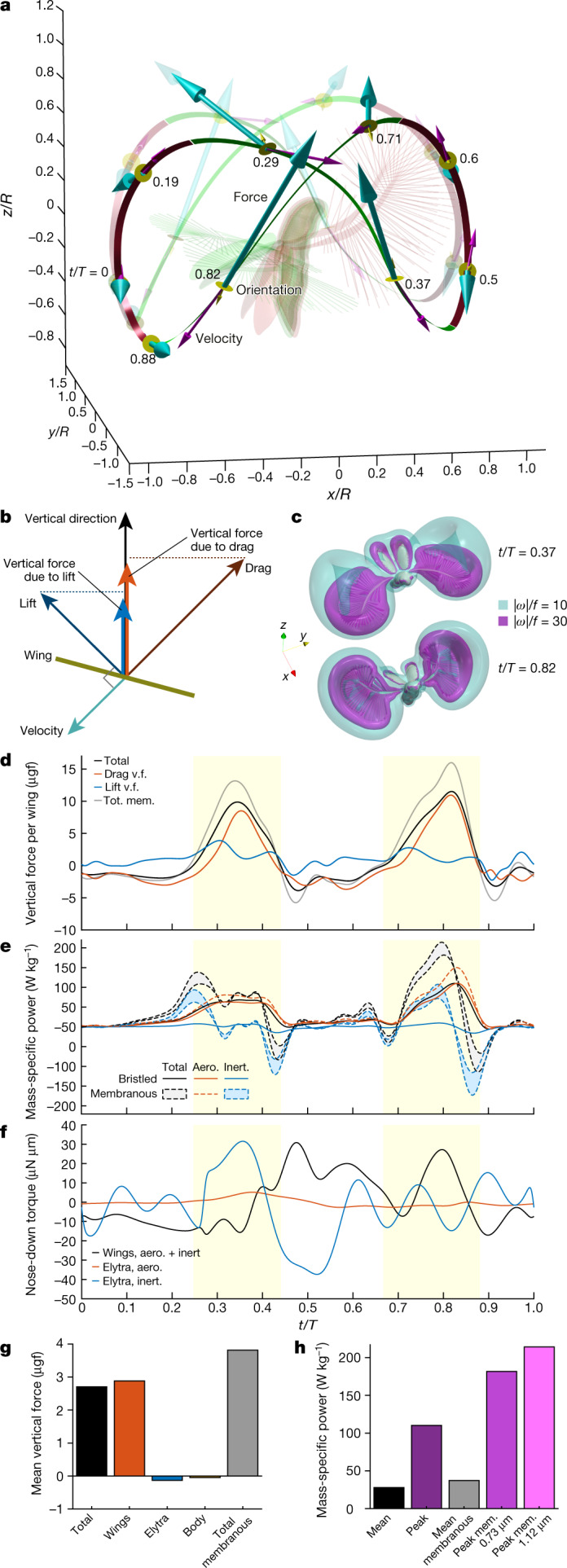
Aerodynamic forces acting on the wings of *P. placentis*. **a**, Wing tip trajectories and direction of total vertical force: downward force (recovery stroke) is shown in green, upward force (power stroke) is shown in red. Posture at *t*/*T* of 0.6 is shown in red, and posture at *t*/*T* of 0.82 is in green. Cyan arrows show aerodynamic force; magenta arrows show wing-tip velocity; yellow discs and arrows show dorsal surface orientation of the wing at nine labelled time instants. Opaque and transparent lines and arrows correspond to right and left wing, respectively. **b**, Vector scheme of forces acting on wing. **c**, Airflow simulation visualized using iso-surfaces of vorticity magnitude (see also Supplementary Video 5). **d**, Vertical aerodynamic force (v.f.) exerted on one wing versus time. Yellow highlighted zones denote the time span of power strokes. Tot. mem., vertical force of membranous wing model. **e**, Body mass-specific aerodynamic (aero.) and inertial (inert.) power, and their sum as the total power. **f**, Pitching torque about centre of mass. The positive direction is nose down. **g**, Contribution of different parts to total aerodynamic force acting on the beetle in the vertical direction, averaged over the wingbeat cycle. **h**, Mean and peak body mass-specific aerodynamic power in computations for bristled and membranous (mem.) wings.

The wide rounded self-intersecting paths of the wing tips and dynamically changing orientation of the wings (Figs. 2c, 3a) maximize the aerodynamic asymmetry between power and recovery strokes. Upon each power stroke, geometrical AoA and wing velocity simultaneously reach their maxima (Fig. 2f). While forces and velocities are anti-aligned (Fig. 3a), their peaks are synchronized (Figs. 3d, 2f). The wing thus first produces an increased upward force as it quickly moves flat-on with net downward displacement and, subsequently, a small downward force while slowly moving edge-on upwards. The near-clap motion reduces the parasite downward force upon recovery^21^. Decomposition of the vertical force exerted on the wing into drag and lift (Methods) is shown in Fig. 3b, d. The vertical force due to drag exhibits greater positive peaks than that due to lift. This is accompanied by extended times of slightly negative drag-based vertical force. In association with these peaks, airflow simulation reveals a pair of strong vortex rings that are typical for drag-producing bodies (Fig. 3c, Supplementary Videos 5, 6). Approximately 32% of the cycle-averaged vertical force results from drag and 68% results from lift, indicating that the beetle benefits from both components. On average, the aerodynamic mechanisms produce bodyweight-supporting lift of 2.7 μg (Fig. 3g) (the beetle’s estimated body mass is 2.4 μg) and a vertical acceleration of 1.0 m s^−2^. The net contribution of body and elytra to the vertical force is negligible (Fig. 3g).

## Stabilizing role of the elytra

The unusually large horizontal and vertical excursion of the wings during flapping poses a peculiar flight dynamics problem. The forces are small during the recovery strokes but the moment arm relative to the centre of mass is large. This results in a pitching moment large enough to overturn the body around its pitching axis (Extended Data Fig. 7, Supplementary Information). To compensate for these moments in synchrony with wing flapping, the insect opens and closes the elytra with large amplitudes (*ψ*_max_ – *ψ*_min_ = 52°) compared to other flying beetles^22–24^. Figures 2e, 3f show that the elytra act as an inertial brake. At times between *t/T* of 0 and 0.3, the wings are raised in a dorsal position and produce nose-up torque. As soon as the wings start their downstroke, the elytra close, causing a nose-down recoil torque on the body. During ventral clapping, the wings produce nose-down torque and the elytra decelerate and reopen. We found that the elytra movements decrease the amplitude of body-pitching oscillation by approximately 50% compared with flight without elytra (Supplementary Information). It is thus likely that the inertial brake observed in *P. placentis* is a feature of ptiliid beetles flying at high wingbeat amplitudes and unique to their peculiar flying style.

## Bristled versus membranous wings

**Extended Data Fig. 1:**
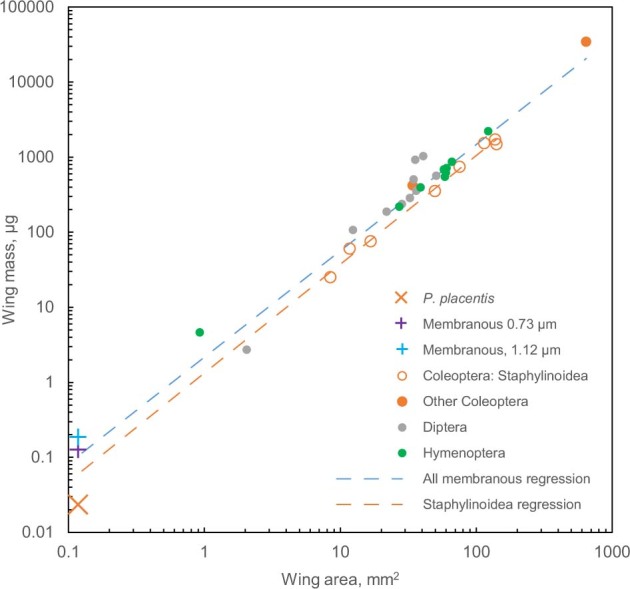
Relationship between wing mass *m*_w_ and wing area *S*. Filled circles show data points for living mass of membranous insect wings from published sources (see Supplementary Information). Punctured circles show dried mass of membranous wings of Staphylinoidea beetles obtained as described in Supplementary Information. The cross indicates calculated weight of the bristled wing of the featherwing beetle *Paratuposa placentis*. Plus signs delimit the estimated mass range of equivalent membranous wings. Blue dashed line is an allometric trend based on all data for membranous wings (*m*_w_ = 2.17 · *S*^1.42^, R^2^ = 0.946). Orange dashed line is an allometric trend based on data for dried wings of Staphylinoidea (*m*_w_ = 1.33·*S*^1.45^, R^2^ = 0.992). The data correspond to mass and area of one wing in Coleoptera and Diptera and of one forewing-hindwing pair in Hymenoptera.

Numerical modelling suggests a wing mass in *P. placentis* of approximately 0.024 µg, which is about 1% of the body mass. By contrast, estimates of the mass of a membranous wing with the same outline amount to 0.13, 0.14 or 0.19 µg, depending on wing thickness (Supplementary Information). These estimates were based on some of the smallest membranous-winged insects, namely the wasp *Trichogramma telengai* (0.73 µm), the beetle *Orthoperus atomus* (0.85 µm), and the beetle *Limnebius atomus* (1.12 µm). *L. atomus* is closely related to the Ptiliidae. The maximum entry in the inertia matrix (*I*_zz_) of the bristled wing is 1,600 µg µm^2^, and for the membranous wings it is 13,800, 16,000 and 20,800 µg µm^2^, respectively. Secondary outgrowths of the bristles are unique to Ptiliidae wings and reduce the wing mass by 44% compared with the bristled wing model with smooth cylindrical bristles at the same drag^25^. The bristled wing architecture with secondary outgrowths thus considerably reduces wing mass compared to a membranous architecture, while maintaining the needed aerodynamic properties. This conclusion is also supported by an allometric analysis of wing mass in differently sized insects (Extended Data Fig. 1, Supplementary Information).

Whereas instantaneous vertical forces generated by the simulated membranous wing outscore the bristled wing of *P. placentis* (Fig. 3d), the latter produces as much as 68% of the mean vertical force of the membranous wing. The vertical force peaks during power strokes produce similar-sized peaks in the mechanical power required for wing actuation. Cycle-averaged power consumption in *P. placentis* is relatively low and amounts to only 28 W per kg body mass, but instantaneous power may reach up to 110 W kg^−1^ at *t*/*T* = 0.82 (Fig. 3e, h) owing to aerodynamic power. The total mechanical power of the bristled wing model (Fig. 3e) remains positive during the entire wingbeat cycle, because low inertia of the wing and high viscous damping of the surrounding air enable continuous energy transfer from the flight apparatus to the wake. No elastic energy storage is required. By contrast, for a membranous wing, the inertial power is similar in peak magnitude to the aerodynamic power. Such a wing requires perfect elastic energy storage to achieve its minimum mean mechanical power of 37 W kg^−1^ and powerful flight muscles to satisfy 180–210 W kg^−1^ peak power requirements (Fig. 3h). The latter computational estimates include aerodynamic added mass effects during wing motion and are detailed in the Supplementary Information.

At low Reynolds numbers, impermeable membranous wings barely outperform leaky bristled wings in generating aerodynamic force. Thus, the small advantage of using a membranous wing is overweighed by the advantage gained in reducing inertial torques and power by minimizing wing mass. This trade-off of energy savings for a small penalty in aerodynamic force generation is available only at Reynolds numbers of about 10 or lower, where sufficiently low leakiness can be achieved with a small number of slender bristles.

## Conclusions and outlook

The findings reported here expand our understanding of the flight mechanics at low Reynolds numbers. In flight, small insects need to produce forces to support their body weight in conditions of high viscous drag on the body and wings. *P. placentis* uses kinematic strategies that maximize wing flapping amplitude but at the potential cost of an increase in inertial power requirements. This is resolved by ptiloptery, an effective structural architecture that serves to reduce inertial costs of wing flapping, making elastic energy storage obsolete and reducing peak mechanical power requirements of the flight muscles. The wingbeat cycle of *P. placentis* is highly functionally divided into power and slow-recovery strokes. The wings thereby produce pronounced high torques that cause the high-amplitude body pitch oscillation. Inertial braking provided by moving elytra represents an ingenious solution to this problem, enhancing posture stability without providing additional forces for flight. In *P. placentis*, these mechanisms improve the temporal distribution of muscle mechanical power requirements and help to maintain aerial performance at an extremely small body size. If this flight style is common for miniature beetles, it may largely explain their worldwide abundance. Further studies of other microinsects with bristled wings will help to reveal the causes of the convergent evolution of ptiloptery during miniaturization in many groups of insects.

## Methods

### Data reporting

No statistical methods were used to predetermine sample sizes. The experiments were not randomized and the investigators were not blinded to allocation during experiments and outcome assessment.

### Material

Adult featherwing beetles (*P. placentis* (Coleoptera: Ptiliidae)) were collected in Cát Tiên National Park, Vietnam, in November 2017. The beetles were collected and delivered to the laboratory together with the substrate for their safety. High-speed video recordings were made on the same day during a few hours after collecting.

### Morphology and morphometry

The material for morphological studies was fixed in alcoholic Bouin solution or in 70% ethanol. Wing structure was studied using a scanning electron microscope (SEM Jeol JSM-6380 and FEI Inspect F50), after dehydration of the samples and critical point drying, followed by gold sputtering. A confocal microscope (CLSM Olympus FV10i-O) and a transmitted light microscope (Olympus BX43) were also used, for which the samples were clarified and microscopic slides were made^26^ (Supplementary Information). Measurements were taken from digital photographs in Autodesk AutoCAD software in ten replications (unless otherwise noted). Body weights and weights of particular body parts were calculated on the basis of three-dimensional reconstructions (Supplementary Information).

### Wing mass and moments of inertia

The volumes of the petiole and membranous part (the blade) of the wing were measured using CLSM image-based geometrical models. Uniform cuticle density 1,200 kg m^−3^ was assumed^27^. The wing mass was obtained by summing up the contributions from the petiole, blade and setae. To calculate the mass of the setae, we first estimated their linear density (0.96 μg m^−1^) using a three-dimensional model^25^ and multiplied it by the length. The petiole and the blade of the wing model have constant thickness without veins. A possible range of the membrane thickness was hypothesized on the basis of measurements in *T. telengai* (Hymenoptera: Trichogrammatidae, body length 0.45 mm), *O. atomus* (Coleoptera: Corylophidae, body length 0.8 mm) and *L. atomus* (Coleoptera: Hydraenidae, body length 1.1 mm), on 0.5 µm thick histological sections obtained by diamond knife cutting using a Leica microtome, after fixation and embedding in araldite. These values are the minimal thicknesses measured in each species. The measurements were performed using an Olympus BX43 microscope. The measurement error of linear dimensions is of the order of magnitude of 1% in the spanwise and chordwise directions and 10% for the thickness. The s.d. of wing cuticle density^25^ is approximately 100 kg m^−3^. This suggests that the overall root sum square error of the wing mass calculation is of about 13%. To evaluate the moments of inertia, surface density of the membranous parts and linear density of the bristles were calculated. The moments of inertia of the individual setae were calculated using the formula for a thin rod at an angle and the parallel axis theorem. The moments of inertia of the membranous parts were calculated using a two-dimensional quadrature rule with the discretization step of 50 μm.

### High-speed recording

Flight of the beetles was recorded in closed 20 × 20 × 20 mm chambers, custom made of 1.0 mm thick microscopic slides and 0.15 mm cover-glass at a natural level of illumination in visible light. There were 20–30 insects in the flight chamber during the recording. For temperature stabilization the flight chamber was chilled by an air fan from the outside. The ambient temperature measured by a digital thermocouple was 22–24 °C; the temperature of the flight chamber was 22–26 °C.

High-speed video recordings were made using two synchronized Evercam 4000 cameras (Evercam) with a frequency of 3,845 FPS and a shutter speed of 20 μs in infrared light (850 nm LED). The high-speed cameras were mounted on optical rails precisely orthogonal to each other and both situated at 0° from the horizon. Two IR LED lights were placed opposite to the cameras and one light above the flight chamber. A graphical representation of the experimental setup can be found in the previous study^2^.

### Measurement of kinematics

**Extended Data Fig. 2:**
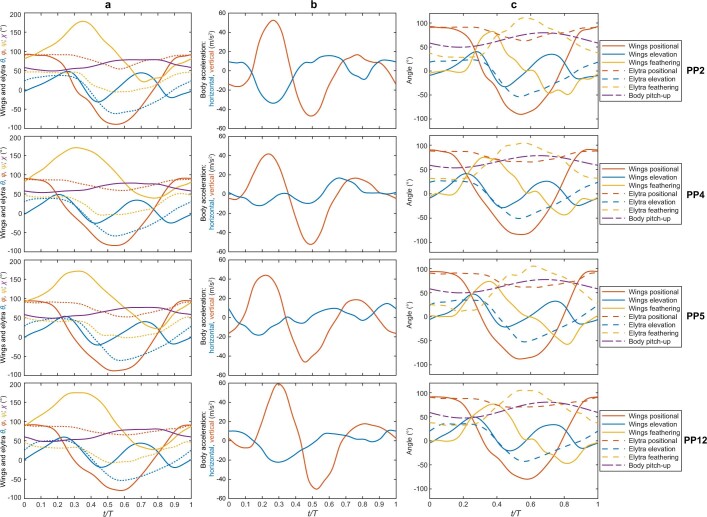
Kinematics descriptions of *Paratuposa placentis* individuals PP2, PP4, PP5, PP12. **a**, Euler angles of wings and elytra and body pitch angle. **b**, Vertical and horizontal components of body acceleration. **c**, Euler angles of wings and elytra and body pitch-up angle between horizontal plane and longitudinal axis of body, prepared for CFD.

**Extended Data Fig. 4:**
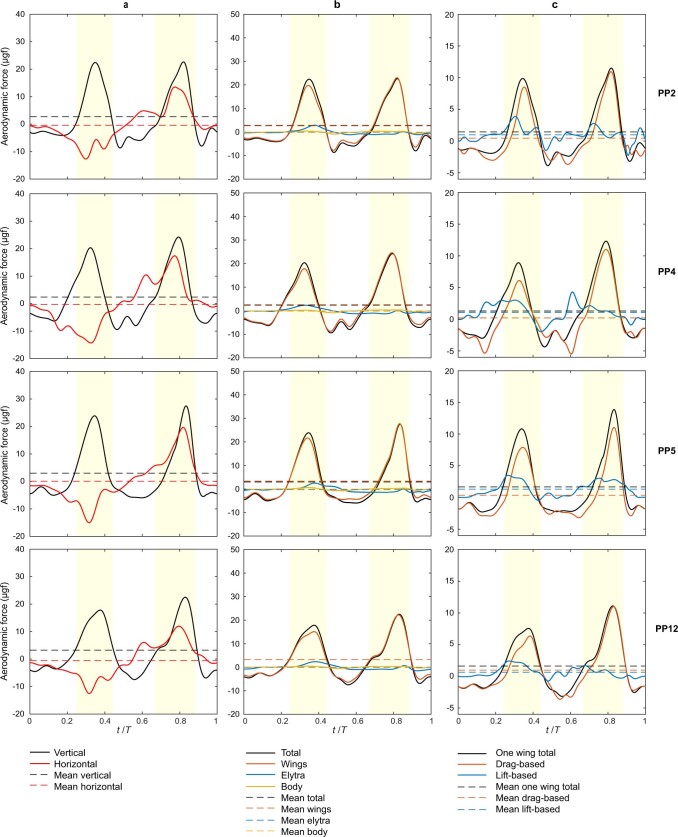
Calculated components of aerodynamic force of *Paratuposa placentis* individuals PP2, PP4, PP5, PP12. **a**, Vertical and horizontal components of the aerodynamic force exerted on the insect. **b**, Total vertical aerodynamic force acting on the insect and its breakdown into the vertical component of the aerodynamic force on the pair of wings, the pair of elytra and the body. **c**, Lift-drag decomposition of the vertical force acting on one wing.

**Extended Data Fig. 5:**
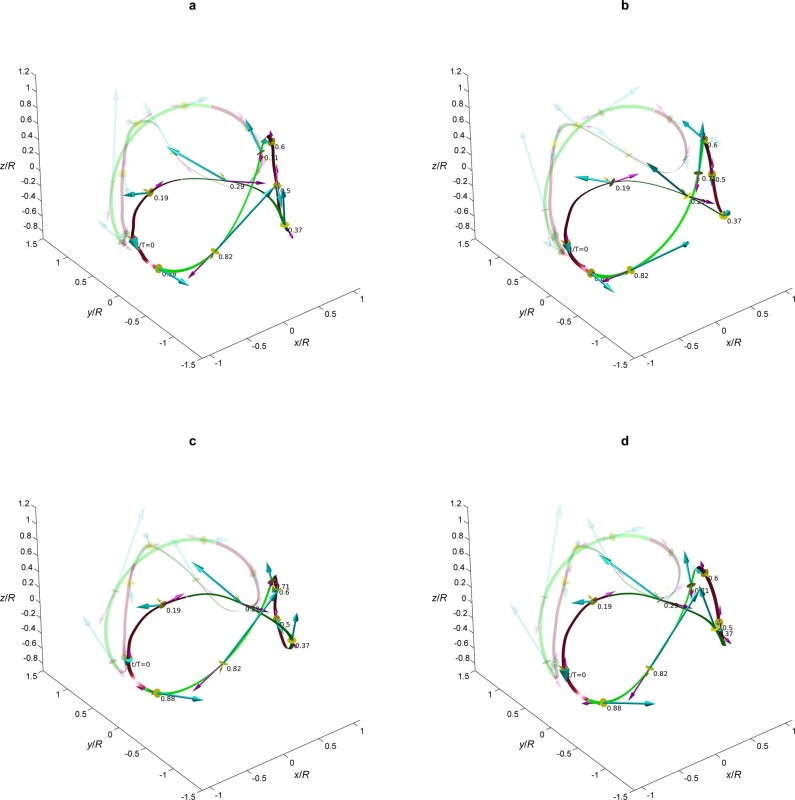
Three-dimensional reconstruction of wing-tip trajectories (continuous lines), aerodynamic force vectors (cyan arrows), velocity vectors (magenta arrows) and wing orientation (yellow circles and arrows) during flight in *Paratuposa placentis* individuals. **a**, PP2. **b**, PP4. **c**, PP5. **d**, PP12. For interactive 3D pdf version, see Supplementary Fig. 9.

**Extended Data Fig. 6:**
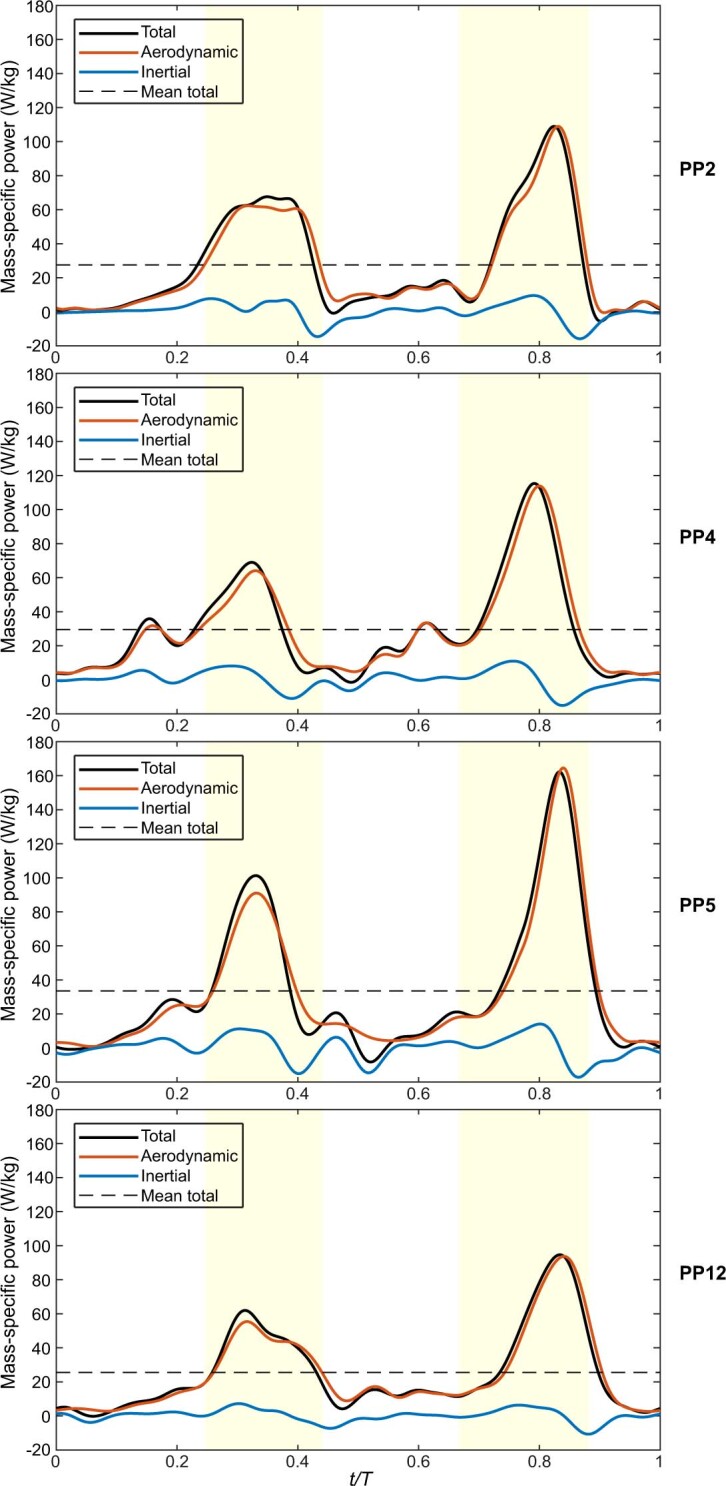
Body-mass-specific mechanical power components of *Paratuposa placentis* individuals PP2, PP4, PP5 and PP12. The mechanical power essentially remains positive through the entire wingbeat cycle period, although occasionally it takes small negative values. The aerodynamic contribution is dominant.

**Extended Data Fig. 7:**
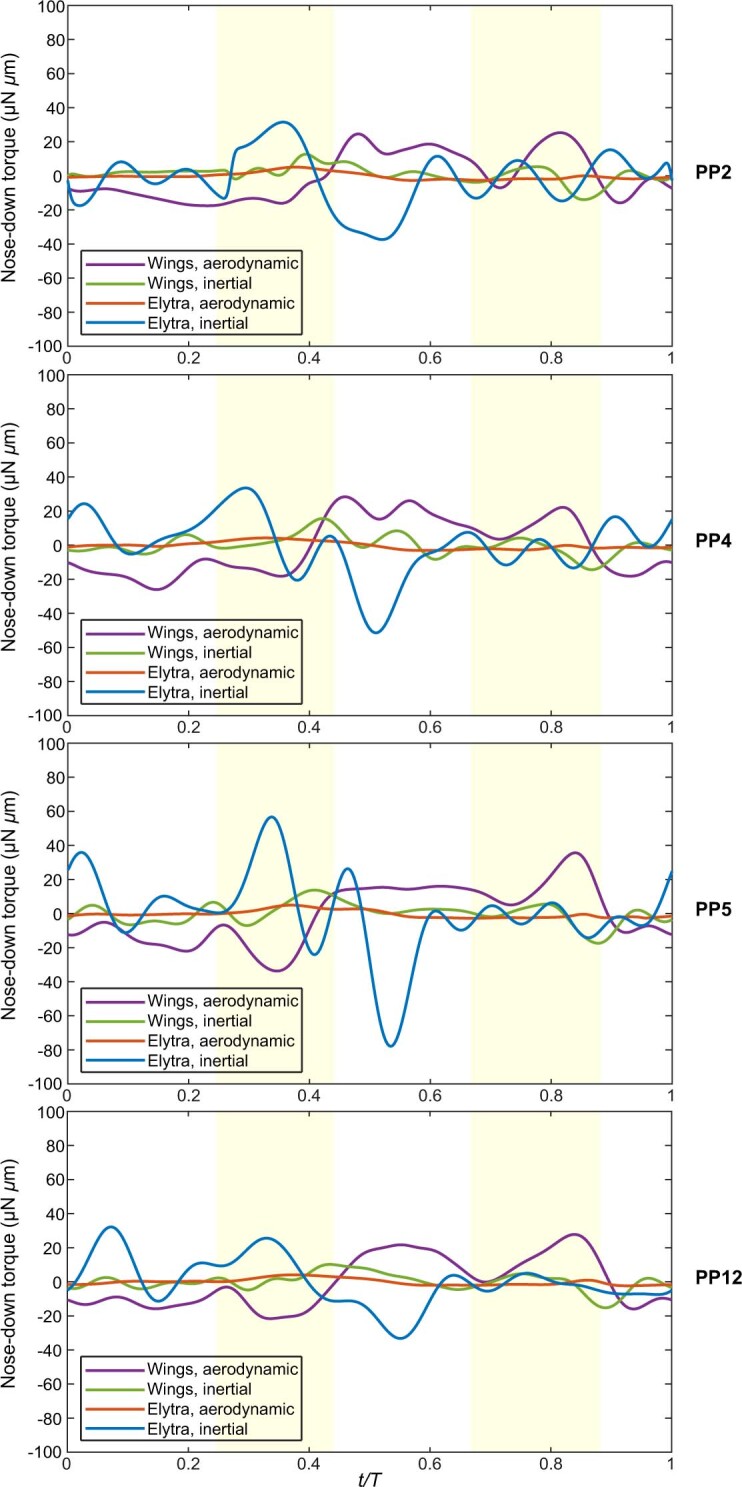
Components of pitching moment acting on *Paratuposa placentis* individuals PP2, PP4, PP5, PP12. Positive direction is nose down.

For analysis, 13 recordings were selected. For four of them (PP2, PP4, PP5 and PP12) we reconstructed the kinematics of body parts in four kinematic cycles for each and performed CFD calculations because the flight of these specimens was especially similar to conventional hovering: relatively slow normal flight with horizontal velocity 0.057 ± 0.014 m s^−1^ (hereafter mean ± s.d.) and 0.039 ± 0.031 m s^−1^ vertical velocity (PP2, PP4, PP5 and PP12). In CFD analysis with the membranous wing model, we selected kinematics of PP2, which does not cross the wings while clapping. This case is convenient for comparing the performance of bristled wings with substitute membranous wings, because it guarantees that the latter do not intersect. The perimeter of the membrane is formed by lines connecting the tips of the bristles (see the previous study^25^ for more information). The descriptions of kinematics and aerodynamics, as well as the illustrations, refer to results obtained for individual PP2. For the results obtained for other specimens, see Supplementary Information and Extended Data Figs. 2, 4–6.

Average wingbeat frequency was calculated as the mean of the wingbeat frequency in all recordings. In each recording, the number of frames was counted in several complete kinematic cycles, 104 cycles in total.

For the mathematical description of the kinematics of the wings and elytra, we used the Euler angles system^28,29^ (Fig. 2b) based on frame-by-frame reconstruction of the location of the insect’s body parts (wings, elytra and body itself) performed in Autodesk 3Ds Max. Three-dimensional models of the body and elytra were obtained by confocal microscope image stacking, and the flat wing model was based on light microscopy photos of dissected wings. We used the rigid flat wing model for reconstruction of the kinematics because the deformations of the wings are minor (Supplementary Information). First, we prepared frame sequences with four full kinematical cycles in each. The frames were then centred and cropped by point between the bases of the wings and then placed as orthogonal projections. Virtual models of body parts were placed into a coordinate system with two image planes. Then we manually changed the position and rotated body parts until their orthogonal projections were superimposed on the image planes. For calculating the Euler angles, a coordinate system was created (Fig. 2a). The *X0Y* plane is a plane parallel to the stroke plane, and intersecting with the base of wing or elytron, which is positioned in the zero point. To determine the position of the stroke plane, we calculated the major axis trend line of the wingtip coordinates instead of the linear trend line^29^, because the wingtip trajectory of *P. placentis* forms a wide scatter plot. Stroke deviation angle (*θ*) and positional angle (*φ*) were calculated from the coordinates of the base and apex. Pitch angle (*ψ*) is the angle between the stroke plane and the chord perpendicular to the line between the base and apex. The body pitch angle (*χ*) is the angle between the stroke plane and longitudinal axis of the body, calculated as the line between the tip of the abdomen and the midpoint between the apical antennomeres. Pitch angle (*β*) of the stroke plane relative to the horizon was also measured.

For flight speed analysis we performed tracking of the centre of the body (middle point between the extreme edges of the head and abdomen) in Tracker (Open Source Physics) in both projections and calculated the instantaneous velocity and its vertical and horizontal components in each frame. The obtained speed values were filtered by loess fitting in R (stats package). The minimum distance between the wingblade tips during bottom claps was also calculated.

### Computational fluid dynamics

Time intervals of low-speed flight with duration longer than four wing beats were selected. The angles *φ*, *θ* and *ψ* of the left wing, right wing and elytra and the body angle χ were interpolated on a uniform grid with time step size Δ*t* = 2.6 × 10^−6^ s. By solving numerically *φ*(*t*) = 0 with respect to *t*, we identified four subsequent wingbeat cycles and calculated the average cycle period *T* and the wingbeat frequency *f* = 1/*T*. We then spline-interpolated the data for each of the four cycles on a grid subdividing the time interval [0, *T*] with step Δ*t*, calculated phase averages, then calculated the average between the left and right wing. This yielded the plots shown in Fig. 2c, d. Constant forward and upward/downward flight velocity was prescribed using the time average values of the loess-filtered time series.

**Extended Data Fig. 8:**
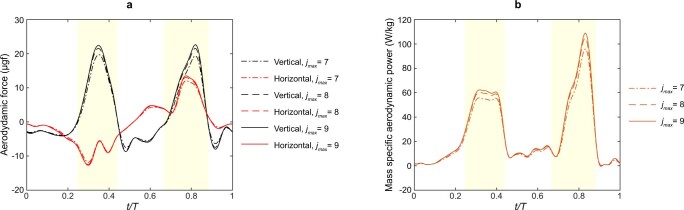
Aerodynamic performance of *Paratuposa placentis* individual PP2 obtained from three different simulations using three different values of maximum refinement level *j*_max_. **a**, Aerodynamic force. **b**, Mass-specific aerodynamic power.

The computational fluid dynamics analysis was performed using the open-source Navier–Stokes solver WABBIT^30^, which is based on the artificial compressibility method to enforce velocity-pressure coupling, volume penalization method to model the no-slip condition at the solid surfaces, and dynamic grid adaptation using the wavelet coefficients as refinement indicators. The flying insect was represented as an assembly of five rigid solid moving parts: the two elytra and the two wings move relative to the body, and the body oscillates about its lateral axis (Supplementary Information). The kinematic protocol is described in Supplementary Information and Extended Data Fig. 2c. The computational domain is a 12*R* × 12*R* × 12*R* cube, where *R* is the wing length, with volume penalization used in combination with periodic external boundary conditions to enforce the desired far-field velocity^30^. The computational domain was decomposed in nested Cartesian blocks, each containing 25 × 25 × 25 grid points. The blocks were created, removed and redistributed among parallel computation processes so as to ensure maximum refinement level near the solid boundaries and constant wavelet coefficient thresholding otherwise during the simulations. The numerical simulations started from the quiescent air condition, continued for a time period of two wingbeat cycles with a coarse spatial grid resolution of Δ*x*_min_ = 0.00781*R* to let the flow develop to its ultimate periodic state, then the spatial discretization size was allowed to reduce to Δ*x*_min_ = 0.00098*R* if the wing was bristled or to Δ*x*_min_ = 0.00049*R* if it was membranous, and the simulation continued for one more wingbeat period to obtain high-resolution results. The air temperature was 25 °C in all cases; its density was *ρ* = 1.197 kg m^−3^ and its kinematic viscosity was *ν* = 1.54 × 10^−5^ m^2^ s^−1^; the artificial speed of sound was prescribed as *c*_0_ = 30.38*fR*, based on an earlier experimental validation^25^. The volume penalization and other case-specific parameter values are provided in Supplementary Information. The CFD simulation accuracy is discussed in Supplementary Information and Extended Data Fig. 8.

### Decomposition of the aerodynamic force of a wing into lift and drag components

The drag component of the total instantaneous aerodynamic force acting on the wing is defined as its projection on the direction of the wing velocity at the radius of gyration. The lift component is defined as a vector subtraction of the total force and the drag component. The total lift and drag force vectors are projected on the vertical (*z*) direction to obtain the time courses shown in Fig. 3d.

### Reporting summary

Further information on research design is available in the Nature Research Reporting Summary linked to this paper.

## Online content

Any methods, additional references, Nature Research reporting summaries, source data, extended data, supplementary information, acknowledgements, peer review information; details of author contributions and competing interests; and statements of data and code availability are available at 10.1038/s41586-021-04303-7.

## Supplementary information

Supplementary Information This file contains Supplementary Sections 1–19, which include Supplementary Figs. 1–9 and Supplementary Tables 1–4.

Reporting Summary

Peer Review File

Supplementary video 1 Flight of *P. placentis* PP2. Synchronized videos in two orthogonal projections.

Supplementary video 2 Flight of *P. placentis* PP4. Synchronized videos in two orthogonal projections.

Supplementary video 3 Flight of *P. placentis* PP5. Synchronized videos in two orthogonal projections.

Supplementary video 4 Flight of *P. placenti*s PP12. Synchronized videos in two orthogonal projections.

Supplementary video 5 Computer rendering of the *P. placentis* PP2 model and airflow visualization. Top left: rendering of the kinematic model used for CFD in lateral projection. Top right: the same model rendering in top projection. Bottom left and right: airflow visualization using iso-surfaces of the vorticity magnitude |*ω*|/*f* = 10 and |*ω*|/*f* = 30 in lateral and top projections. The laboratory reference-frame axes *x*, *y* and *z* are shown as red, yellow and green arrows, respectively.

Supplementary video 6 Computer rendering of the *P. placentis* PP5 model and airflow visualization. Top left: rendering of the kinematic model used for CFD in lateral projection. Top right: the same model rendering in top projection. Bottom left and right: airflow visualization using iso-surfaces of the vorticity magnitude |*ω*|/*f* = 10 and |*ω*|/*f* = 30 in lateral and top projections. The laboratory reference-frame axes *x*, *y* and *z* are shown as red, yellow and green arrows, respectively.

## Figures and Tables

**Extended data Fig. 3:**
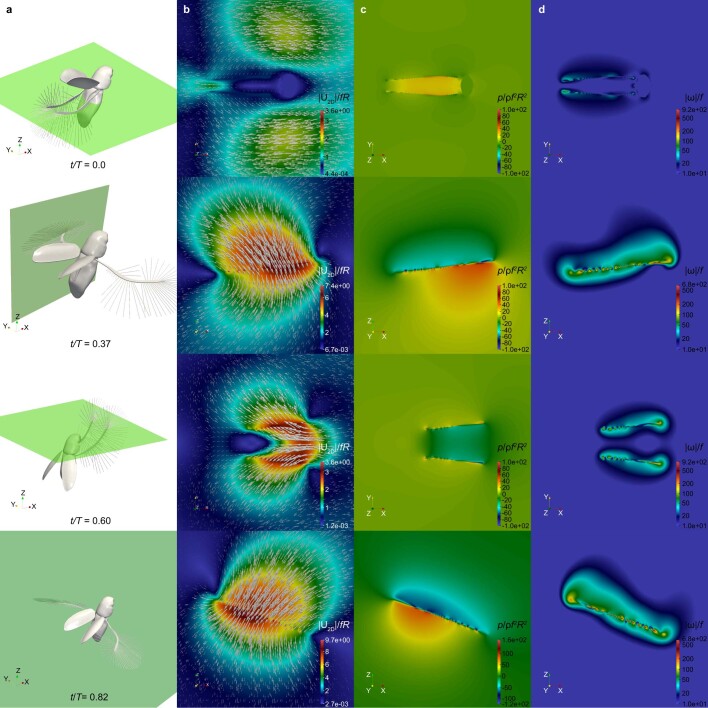
CFD simulation of the flight of *Paratuposa placentis*: visualization of the flow using two-dimensional slices in the 3rd simulation cycle. Time instances are *t/T* = 0.0 (dorsal recovery stroke) *t/T* = 0.37 (first power stroke), *t/T* = 0.60 (ventral recovery stroke) and *t/T* = 0.82 (second power stroke), from top to bottom. **a**, Planes perpendicular to wings. **b**, Velocity vectors (arrows) and velocity magnitude (background colouring), showing two-component in-plane velocity with the third vector component omitted for clarity. **c**, Pressure. **d**, Magnitude of three-dimensional vorticity with colouring in logarithmic scale.

**Extended Data Fig. 9:**
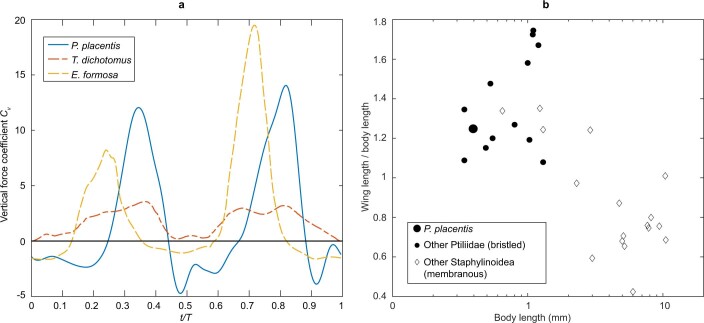
Comparison of *Paratuposa placentis* and other insects that have bristled or membranous wings. **a**, Time variation of the vertical force coefficient in three different species. Data for the large rhinoceros beetle *Trypoxylus dichotomus* and for the tiny chalcid wasp *Encarsia formosa* are adapted so that the cycle begins with the downstroke. The force coefficient is defined as *C*_*V*_ = 2*F*_*V*_/*ρ*(2*ΦR*_*g*_*f*)^2^*S*, where *F*_*V*_ is vertical force, *ρ* is air density, *Φ* is flapping amplitude, *R*_*g*_ is wing geometric radius of gyration, *f* is flapping frequency, and *S* is wing area. Note that the bristled wings of *Encarsia formosa* were modelled as impermeable solid plates. For additional information, see Supplementary Information. **b**, Wing length relative to body length.

**Extended Data Fig. 10:**
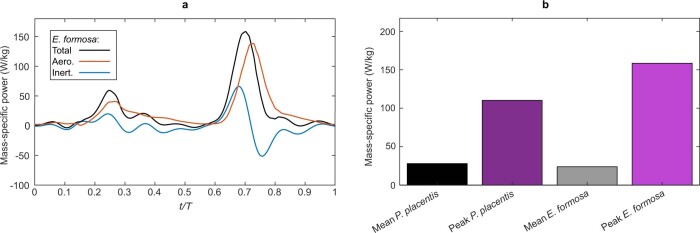
Comparison of *Paratuposa placentis* and *Encarsia formosa*. **a**, Time variation of body-mass-specific mechanical power. **b**, Mean (averaged over wingbeat cycle) and peak (maximum over the cycle) body-mass-specific mechanical power.

## Data Availability

Extended data sets and raw data are available in an Open Science Framework repository (https://osf.io/v3wrk/).

## Code availability

Computational fluid dynamics simulations were performed using an open-source code WABBIT, which can be downloaded from GitHub (https://github.com/adaptive-cfd/WABBIT) and has been described in detail elsewhere^30^.
