# Supplementary material for: Novel flight style and light wings boost flight performance of tiny beetles
Source: Nature. 2022 Jan 19;602(7895):96–100. doi: 10.1038/s41586-021-04303-7 (PMC8810381; doi:10.1038/s41586-021-04303-7)
Supplement: Supplementary file 1 — This file contains Supplementary Sections 1–19, which include Supplementary Figs. 1–9 and Supplementary Tables 1–4. [file 41586_2021_4303_MOESM1_ESM.pdf]

nature portfolio
https://doi.org/10.1038/s41586-021-04303-7

Supplementary information

Novel flight style and light wings boost flight performance of tiny beetles

In the format provided by the
authors and unedited

Novel flight style and light wings boost flight performance of tiny beetles

Supplementary information

Sergey E. Farisenkov[1,*], Dmitry Kolomenskiy[2,3*], Pyotr N. Petrov[1], Thomas Engels[4], Nadezhda A. Lapina[1], Fritz-Olaf Lehmann[4], Ryo Onishi[2], Hao Liu[5], Alexey A. Polilov[1,6]

[1]Department of Entomology, Biological Faculty, Lomonosov Moscow State University, Leninskie gory 1-12, Moscow, 119234 Russia

[2]Global Scientific Information and Computing Center, Tokyo Institute of Technology, 2-12-1 O-okayama, Meguroku, Tokyo, 152-8550 Japan

[3]Skoltech Center for Design, Manufacturing and Materials, Skolkovo Institute of Science and Technology, Bolshoi blvd. 30-1, Moscow, 121205 Russia

[4]Department of Animal Physiology, Institute of Biological Sciences, University of Rostock, Albert-Einstein-Str. 3, 18059 Rostock, Germany

[5]Graduate School of Engineering, Chiba University, 1-33 Yayoi-cho, Inage-ku, Chiba-shi, Chiba, 263-8522 Japan

[6]Joint Russian-Vietnamese Tropical Research and Technological Center, Southern Branch, Hẻm Số 3 Đường 3 Thàng 2, Ho Chi Minh, 70000 Vietnam.

Table of contents

Titles and legends for SI videos 1
Section 1 – Body size measurements 2
Section 2 – Wing morphometrics 2
Section 3 – Allometric analysis of wing mass 2
Section 4 – Wing deformations 2
Section 5 – 3D reconstruction of kinematics 3
Section 6 – On the similarity with rowing and breaststroke 3
Section 7 – Calculation of the geometric radius of gyration 3
Section 8 – Building 3D models for CFD 4
Section 9 – Calculations of mass and moments of inertia for body, wing and elytron 5
Section 10 – Kinematic model for CFD 5
Section 11 – CFD simulation setup 6
Section 12 – Details of the flow around the setae 7
Section 13 – CFD results 8
Section 14 – Mechanical power calculation 10
Section 15 – The effect of elytra on body pitch oscillation 10
Section 16 – Numerical convergence 10
Section 17 – On the fluid-dynamic added mass effect 11
Section 18 – Comparison with larger beetles and tiny insects of other orders 11
Section 19 – Ptiloptery and the original flying style help beetles to fly faster 11
References 11

Corresponding authors: Sergey E. Farisenkov, Alexey A. Polilov

Emails: farisenkov@entomology.bio.msu.ru, polilov@gmail.com

*These authors contributed equally.

Titles and legends for SI videos

Supplementary video 1. Flight of *Paratuposa placentis* PP2.
Synchronized videos in two orthogonal projections.

Supplementary video 2. Flight of *Paratuposa placentis* PP4.
Synchronized videos in two orthogonal projections.

Supplementary video 3. Flight of *Paratuposa placentis* PP5.
Synchronized videos in two orthogonal projections.

Supplementary video 4. Flight of *Paratuposa placentis* PP12.
Synchronized videos in two orthogonal projections.

Supplementary video 5. Computer rendering of the *Paratuposa placentis* PP2 model and air flow visualization.
Upper left: rendering of the kinematic model used for CFD in lateral projection. Upper right: the same model rendering in top projection. Bottom left and right: air flow visualization using iso-surfaces of the vorticity magnitude $|\omega|/f = 10$ and $|\omega|/f = 30$ in lateral and top projections. The laboratory reference frame axes x, y and z are shown as the red, yellow and green arrows, respectively.

Supplementary video 6. Computer rendering of the *Paratuposa placentis* PP5 model and air flow visualization.
Upper left: rendering of the kinematic model used for CFD in lateral projection. Upper right: the same model rendering in top projection. Bottom left and right: air flow visualization using iso-surfaces of the vorticity magnitude $|\omega|/f = 10$ and $|\omega|/f = 30$ in lateral and top projections. The laboratory reference frame axes x, y and z are shown as the red, yellow and green arrows, respectively.

Section 1 – Body size measurements

It was not possible to measure the body mass of *Paratuposa placentis* Deane, 1931 directly, because the total mass of all specimens was less than the lower capacity of the laboratory balance. Therefore, *P. placentis* body mass was calculated based on the body mass of the closely related beetle *Primorskiella* sp., which has a similar body size and proportions (Supplementary Fig. 1). A total of 179 *Primorskiella* sp. specimens were immobilized by carbon dioxide and weighed on a Satrogosm MB210-A laboratory balance (Satrogosm LLC, Russia). Their total weight was 0.82 mg, and therefore the mean weight of one specimen was 4.58 µg. Confocal stacks of 10 specimens of each species were obtained by Olympus FV10i-O (Olympus Corporation, Japan), using autofluorescence in channels of 488 and 559 nm lasers. The samples were depigmented using hydrogen peroxide solution (Dimethyl sulfoxide + 100% EtOH + 30% H_2O_2 in proportions 1 + 3 + 1, respectively) for 1–5 days at a temperature of 37 °C. Then the samples were dehydrated in ethanol solutions of increasing concentrations (80, 95, 100, and 100%) and clarified in BABB (Benzyl Alcohol + Benzyl Benzoate in proportions 1 + 2) for 24 hours. Preparations with the samples were made using two cover glasses and FEP ring spacers. Body reconstructions were built from confocal stacks in Bitplane Imaris using Surface function and automatic image segmentation (Supplementary Fig. 1). Volumes of body reconstructions were measured in the statistical module of the Imaris program. The body volume of *Primorskiella* sp. was 5.33 ± 0.16 nL (hereinafter M ± s.d.); its density was therefore 0.86 ± 0.02 µg/nL. The body volume of *P. placentis* was 2.83 ± 0.22 nL. Assuming that the body density of both species is equal, the body mass of *P. placentis* is 2.43 ± 0.19 µg.

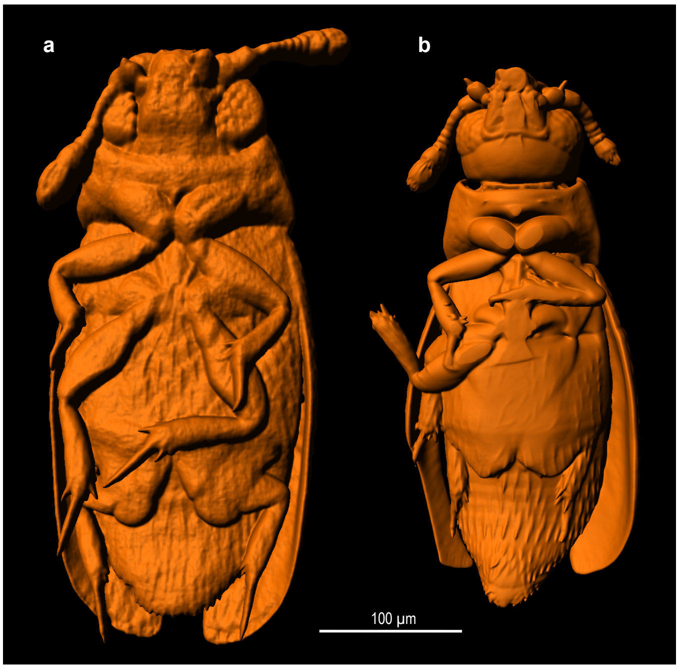

Supplementary Figure 1. 3D reconstruction of featherwing beetles. a, *Primorskiella* sp. **b,** *Paratuposa placentis.*

The body length of each living specimen was measured in high-speed recordings as the distance between the anterior edge of the head and the posterior edge of the abdomen. The three-dimensional body length was calculated based on lengths it two orthogonal projections. The values in frame sequences (from 21 to 106) were averaged for each recording. The average body length of all recorded specimens was 395 ± 21 µm. The body length measured by confocal stacks was 375 ± 1 2 µm. Thus, the body length of living specimens was higher than that of fixed ones (Mann–Whitney U test, $p < 0.01$), probably because of contraction of intersegment abdominal muscles after fixation. In this connection, we calculated the body mass of the recorded beetles taking into account the changes in their body shape. The body mass of the recorded specimens in kg was estimated using the formula $m_b = \rho K_{iso} l_b^3$, where $K_{iso} = 45.95$, $\rho = 0.86$ kg/m^3 and l_b is the in-flight body length in m.

Section 2 – Wing morphometrics

Ten dissected wing preparations were manufactured according to the protocol described in our previous study[1]. The morphometric characteristics (wing length, total wing area and area of the membranous part plus petiole) were measured in Autodesk AutoCAD, using images taken with a light microscope.

Section 3 – Allometric analysis of wing mass

In this section, we compare our wing mass calculations with available mass measurement data. Extended Data Fig. 1 shows the wing mass m_w plotted against the wing area S for a variety of insects. Direct measurements are scarce for wings of areas less than 10 mm^2. Therefore, the allometric trends are extrapolated down to $S = 0.118$ mm^2 in order to compare with *P. placentis*. Extrapolation of the general trend yields $m_w = 0.115$ µg, which is close to the estimate obtained assuming a 0.73 µm-thick membrane. The value based on the trend for desiccated Staphylinoidea wings is $m_w = 0.066$ µg, which is expectedly lower, but not as low as m_w of the bristled wing.

Sources[2,3,4,5,6] report multiple measurements of individual samples. The points in Extended Data Fig. 1 are the corresponding averages for each species and each source. In addition, in the cases where the wing data are presented in a dimensionless form[2], we recovered the dimensional parameters before calculating the respective averages. For *Glossina* spp. (tsetse flies)[7], allometric relationship is reported for m_w as a function of 2S. Extended Data Fig. 1 contains only two extreme points on it that correspond to the smallest and the largest tsetse fly wings, respectively. For the small wasp *Nasonia vitripennis* (Walker, 1836), the wing area measurements have been published, but the wing mass data remained unpublished[8]. The *Drosophila* wing mass[9] is a result of a calculation rather than a direct measurement. All points in Extended Data Fig. 1 correspond to the area and the mass of one wing, except for the Hymenoptera species, in which case a point corresponds to a single forewing-hindwing pair.

In addition to the published data, measurements of the wing area and mass of seven species of Staphylinoidea (the superfamily containing Ptiliidae) were taken: Staphylinidae: *Lordithon lunulatus* (Linnaeus, 1760), *Philonthus* sp. and *Aleochara* sp.; Silphidae: *Nicrophorus vespillo* (Linnaeus, 1758), *N. investigator* (Zetterstedt, 1824), *N. humator* (Gleditsch, 1767) and *Oiceoptoma thoracicum* (Linnaeus, 1758). The material was taken from the collection of the Department of Entomology, Faculty of Biology, Lomonosov Moscow State University. Dry wings were weighed on a Satrogosm MV210-A laboratory balance (Satrogosm LLC, Russia) at a temperature of 25 °C and a relative humidity of 50%. Wings of the larger beetles were weighed separately; small wings were weighed in several pieces. To measure the wing area, temporary microscopic preparations of the straightened wings in glycerol were made; they were photographed on an Olympus SZX16 microscope with a Canon 1000D camera. The area measurement was performed in the same way as described in SI 2.

Section 4 – Wing deformations

For evaluation of transverse wing deformations, we selected frames that showed the wing in lateral view during power strokes (Supplementary Fig. 2a): 12 upstroke frames and 10 downstroke frames. Since at these moments the speed and angle of attack and therefore drag are near the local maximum, the wing is under the highest lateral loads and most deformed.

In lateral view three points can be identified on the wing: the base (P_1), the wingtip (P_5) and the point on the border between the petiole and the membrane (P_3). P_3 was marked at the border that divides the wing into the distal part (membrane plus bristles) and the proximal part (petiole). We marked point P_2 in the geometric middle of the petiole and point P_4 in the geometric middle of the distal part (Supplementary Fig. 2b, c).

The flexion of the petiole was measuredas the angle λ_1 between sections P_1P_2 and P_1P_3. The flexion of the distal part is the angle between P_3P_4 and P_3P_5. The total wing flexion is the angle λ between P_1P_3 and P_1P_5. The deformation range of the wing is the sum of the average upstroke and downstroke λ.

The deformation range of the distal part of the wing is 7.6°; the petiole deforms to a smaller degree, on average within 3.5° (Supplementary Table 1). The average deformation range of the wing is 16.5°. The wing has an S-shaped profile during the downstroke and a J-shaped profile during the upstroke (Supplementary Fig. 2b). Resolution did not allow us to measure deformations of the bristles separately, because the bases of the bristles could not be identified. But it was visible that the flexion between P_4P_5 was less than between P_3P_4, so the deformation of the bristles was smaller than deformations of the membranous part of the wing.

Wing drag makes up most of the resulting aerodynamic force and is proportional to the projection of the wing onto the plane perpendicular to its instantaneous velocity. Because the area and velocity of the petiole are minor, its contribution to drag can be neglected. The area of the projection and the drag of the rest of the wing depend on the flexion of the distal part and are proportional to $\cos\lambda_2$, which varies within 1% of the estimated resting position. Such a small influence on the aerodynamics allows us to ignore deformations in kinematics reconstructions and CFD calculations and use rigid wing models.

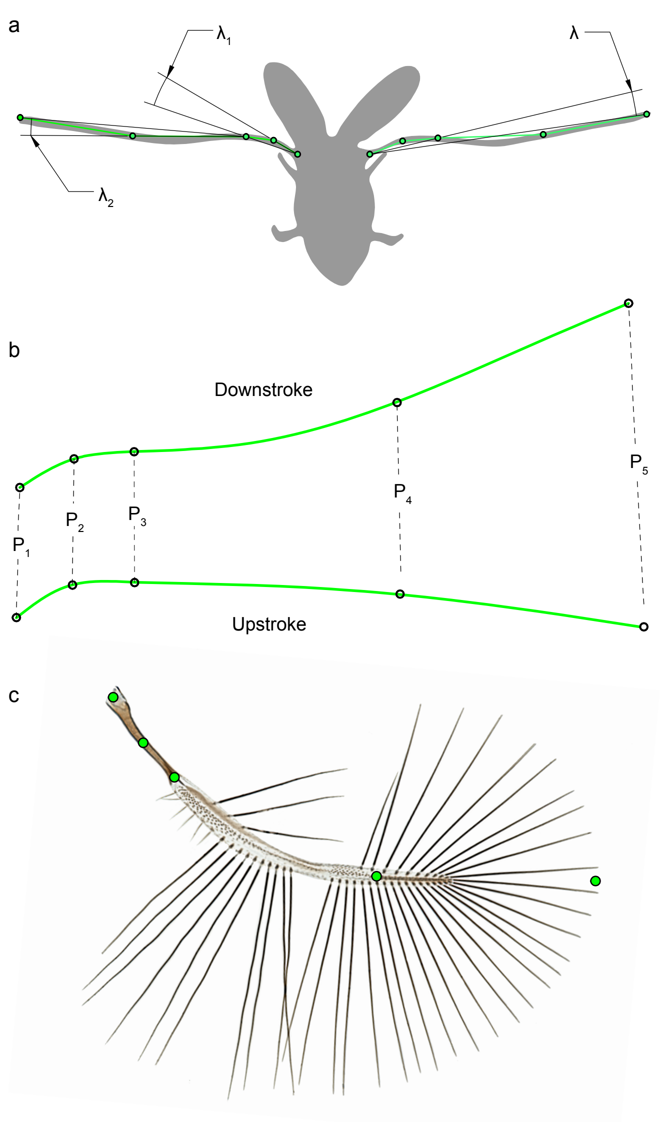

Supplementary Figure 2. Deformations of the longitudinal wing section during downstroke and upstroke. a, Measurement of deformation angles of wing (λ), petiole (λ_1) and distal part of wing (λ_2). **b,** Averaged shape of longitudinal wing section during downstroke and upstroke; location of landmarks P_1–P_5, lateral view. **c,** Location of marks P_1–P_5, dorsal view.

Supplementary Table 1. Mean values of wing deformation angles during downstroke and upstroke.

Deformation angle	λ_1 (°)		λ_2 (°)		λ (°)	
	Mean	s.d.	Mean	s.d.	Mean	s.d.
Upstroke	14.0	2.7	2.4	1.0	18.2	5.7
Downstroke	10.5	2.1	5.2	2.6	1.7	7.4
Range	3.5		7.6		16.5	

Section 5 – 3D reconstruction of kinematics

The time courses of the Euler angles of the wings and elytra and body pitch angle of individual beetles PP2, PP4, PP5 and PP12 are shown in Extended Data Fig. 2a. There are minor differences between individuals. Vertical and horizontal components of instantaneous acceleration strongly vary depending on the stage of the cycle (Extended Data Fig. 2b). Vertical acceleration is synchronized with the time course of the lift force.

The wings clap dorsally and near-clap ventrally. During the ventral near-claps after downstroke, the minimal distance between tips of the membranous parts of the wings of beetles PP2, PP3, PP5, PP6, PP8, PP10 and PP12 is 0.40 ± 0.23 body lengths. It correlates negatively with the vertical component of the instantaneous body velocity (Spearman $r_s = -0.78$, $p < 0.05$, $n = 25$). It can be assumed that with increasing vertical velocity, viscous drag force decreases the lift, and the insect compensates this drag through more pronounced wing clapping.

Section 6 – On the similarity with rowing and breaststroke

The propulsive mechanism of *P. placentis* may be compared with rowing, as the term "power stroke" suggests: the insect wing and the oars of a boat alike produce useful thrust in short pulses, interlaced with the recovery phases. While this analogy is illustrative, it is important to mind its limitations. When the oar is submerged, it can impart a much greater momentum than when it is in air, because water is a thousand times denser than air. Thus, the surrounding medium density stratification is exploited. By contrast, the wings of *P. placentis* vary in orientation and speed to generate more useful force during power stroke than parasite counter-acting force during recovery. But the resulting variation in the momentum is not as dramatic as in the case of a boat.

The *P. placentis* flight style shares more similarity with underwater breaststroke: the appendages extend during the power stroke to maximize the drag and contract during the recovery stroke to minimize it, respectively. The breaststroke gait is also used by microorganisms such as the green algae *Chlamydomonas*.

Another important difference is that the wings of *P. placentis* should generate a large enough aerodynamic force to support the body weight. Therefore, the wing beat frequency is relatively high: even during the slow forward flight as considered in the present study, the body velocity is less than 10% of the maximum velocity at the geometric radius of gyration (R_g) during power stroke. The reported maximum flight speed[11] of 0.31 m/s is less than 50% of the same maximum velocity at R_g. Therefore, the instant center of rotation of the wing is located near the hinge rather than near the wing apex. For a rowing boat, this would not be a typical operating regime. For microorganisms such as *Chlamydomonas*, it is typical[12].

Section 7 – Calculation of the geometric radius of gyration

It is customary to define dimensionless aerodynamic properties of flapping wings, such as the Reynolds number, with reference to the geometric radius of gyration determined from the second moment of area[2,13]. This is natural for membranous wings that generate aerodynamic forces almost exclusively due to differential pressure at the surface, which results in dynamical similarity scaling with the projected area of the wing. For a bristled wing, however, about half of the normal force is due to the shear stress[14] that depends on the total surface area.

Considering that bristled wings function as leaky paddles producing between 66 and 96% of the aerodynamic drag force of equivalent membranous wings[14], it is reasonable to use the equivalent membranous wing projection for calculating the radius of gyration. This approach results in dimensional numbers that are in concord with those known for

membranous wings, such that they can be compared directly without amendment for the reference area difference.

The radius of gyration R_g in the present study was calculated as follows. Note that this is a geometrical quantity, i.e., it does not take into account the mass distribution. The equivalent membranous wing projection on the x_w-y_w plane defines the set of points Ω_w lying inside the wing contour (Supplementary Fig. 4). The indicator function $I_{\Omega w}$ is sampled on a uniform grid covering a rectangular region of x_w from -0.6R to 0.4R and y_w from 0 to 1.1R with step 0.001R. This produces an array of values equal to 1 in the interior of the contour and 0 otherwise. The geometric radius of gyration is evaluated as

$$R_g = \left(\iint_{\Omega_w} y_w \mathrm{I}_{\Omega_w} dx_w dy_w \Big/ \iint_{\Omega_w} I_{\Omega_w} dx_w dy_w \right)^{1/2} \approx \left(\sum_i \sum_j y_{wj} \mathrm{I}_{\Omega_w}(x_{wi}, y_{wj}) \Big/ \sum_i \sum_j I_{\Omega_w}(x_{wi}, y_{wj}) \right)^{1/2} = 0.63R \quad (1)$$

The velocity at the radius of gyration is the velocity of a point on the longitudinal axis of the wing at the distance R_g from the hinge. The values shown in Fig. 2f and Supplementary Table 3 correspond to the magnitude of the velocity in the laboratory reference frame.

Section 8 – Building 3D models for CFD

The insect is represented in our 3D models as an assembly of five rigid solid parts: two elytra, two wings, and a body. Supplementary Fig. 3 shows the bristled model of the left wing. It is similar to the model used in the earlier rotating wing simulations[14]. The main difference is in the out-of-plane deviation, which is substantial in the proximal part of the blade, according to the confocal microscope data. The distal part of the wing is essentially planar. The wing length is denoted as R. In the aerodynamic calculation, the bristles are modelled as circular cylinders with diameter b = 0.00388R, which is justified by the prior work[14]. The central blade thickness is h_b = 0.008R according to the confocal microscope data. The right wing used in the simulations differs from the left wing by a small rotation of the bristles in the x_w-y_w plane around their respective base points, so that the bristle tips deflect by a distance four times as large as the bristle diameter b. This is sufficient to prevent collision between the wings when they clap.

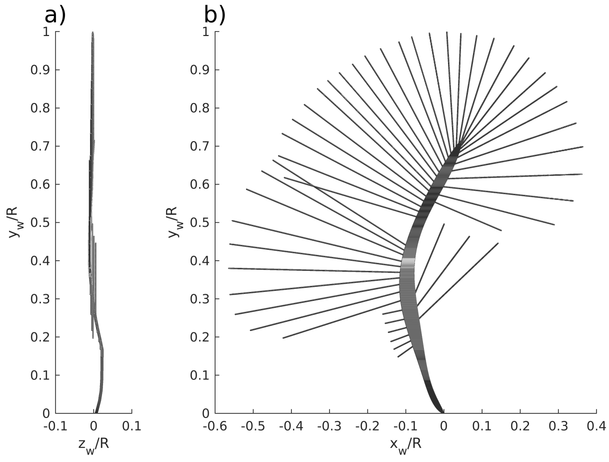

Supplementary Figure 3. Orthographic projection view of the bristled wing model (left wing). Distances are scaled by the wing length R. **a,** View from the trailing edge side. **b,** Morphologically dorsal view.

Supplementary Fig. 4 shows the membranous wing model. It is constructed by sealing the gaps between the bristles. This is similar to gluing adhesive tape sheets in prior experiments[14]. To simplify the geometrical processing, the membranous wing is made flat. It is only used in conjunction with the wing kinematics of individual PP2. The latter is such that the wings do not intersect when they clap. In the aerodynamic calculation, the wing thickness at any point is equal to the blade thickness h_b. This enables computing with the minimum discretisation step Δx_{min} twice as large as in the bristled wing aerodynamic simulations. The left and the right membranous wings are mirror images of each other.

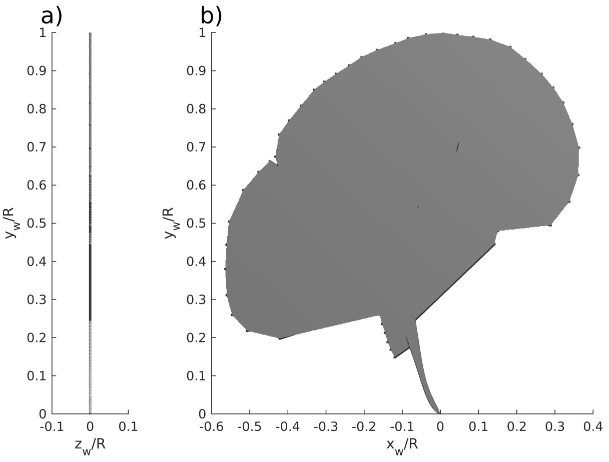

Supplementary Figure 4. Orthographic projection view of the membranous wing model. Distances are scaled by the wing length R. **a,** View from the trailing edge side. **b,** Morphologically dorsal view.

The two elytra are also mirror images of each other. The volumetric models are based on confocal microscope data (Supplementary Fig. 5). However, the proximal part has been modified to avoid intersection with the body. For the elytra and for the wings alike, the centre of rotation is at the origin of the respective local coordinate system. The thickness in the aerodynamic calculation is uniform, h_e = 0.008R. Note that the assumed uniform thickness of the elytra and the wings in the aerodynamic geometrical models described here is, in general, different from the thickness in the inertia calculations.

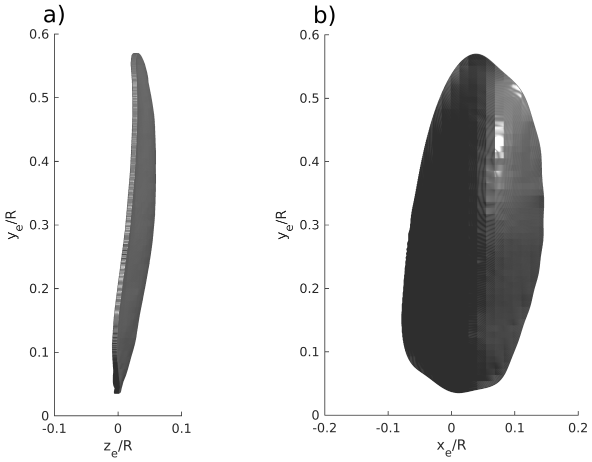

Supplementary Figure 5. Orthographic projection view of the left elytron model. Distances are scaled by the wing length R. **a,** View from the trailing edge **b,** Morphologically dorsal view.

The body model (Supplementary Fig. 6) is also based on information from the confocal microscope, and it is stored as a mesh surface. Note that it is not perfectly bilaterally symmetric. It is also noteworthy that, while the position of the hinge points in the model closely matches the attachment location of the wings measured from SEM images, the position of the elytron hinges has been slightly offset to prevent intersection between the elytra and the wings as they undergo rigid solid body rotation.

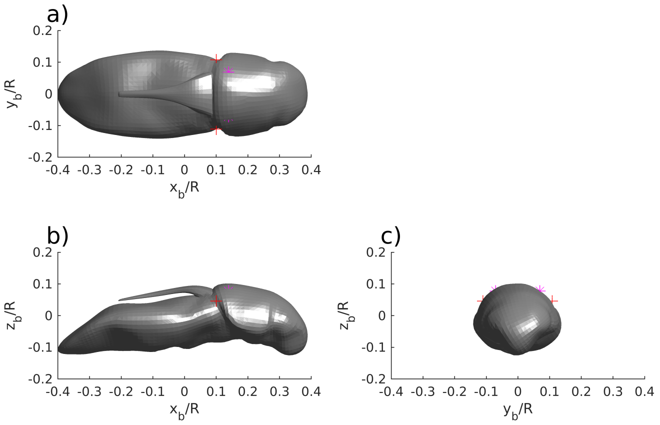

Supplementary Figure 6. Orthographic projection views of the body model. The red "+" signs show the wing hinge points. The magenta asterisks show the virtual hinge points of the elytra. Distances are scaled by the wing length *R*. **a,** Dorsal view. **b,** Lateral view. **b,** Front view.

Section 9 – Calculations of mass and moments of inertia for body, wing and elytron

The second moment of inertia of the body about the pitching axis was calculated in Autodesk Inventor Professional 2019 (Autodesk Inc., U.S.A.) using the CAD model of the body shown in Supplementary Fig. 6. The surface model described above, after geometric simplification, was "converted to base feature" of Autodesk Inventor and the inertial properties were evaluated assuming uniform density distribution. In combination with the body mass scaling formula, we obtained the following isometric scaling for the moment of inertia in kg·m^2 as a function of the body length in m: $J_b = 2.14 l_b^5$. After appropriate rescaling, we obtained the principal moments of inertia: $J_{bxx} = 0.30 \cdot 10^{-17}$ kg·m^2 about the anterior-posterior axis, $J_{byy} = 2.06 \cdot 10^{-17}$ kg·m^2 the about the lateral axis and $J_{bzz} = 2.15 \cdot 10^{-17}$ kg·m^2 about the dorsal-ventral axis. These values are scaled to the body length 0.37 mm and mass 2.43 µg, which correspond to individual PP2 and coincide with the statistical averages. Moments of inertia of individuals PP4, PP5 and PP12 are calculated by rescaling the data for PP2 in proportion with the body length to the power of 5.

The wing mass and moments of inertia were calculated by summing up the contributions from the central membranous part (blade) and the peripheral setae (bristles). The mass of the blade (0.0162 µg) was evaluated as a product of the volume enclosed by the SEM surface model obtained by confocal microscopy stacking ($1.346 \cdot 10^{-5}$ mm^3) and the cuticle density. The latter was taken as 1200 kg/m^3 in all calculations related to the wings and the elytra[15]. To calculate the mass of the setae, we first estimated their linear density (0.96 µg/m) using a 3D model consisting of a short cylindrical segment and a few secondary outgrowth elements. The same model was used in our previous study[14] to select the aerodynamically equivalent circular cylinder section. The mass of each seta was then found by multiplying its length by the linear density. By summing up the masses of all setae, we determined the mass of the peripheral bristled part of the wing (0.0073 µg for PP2). Thus, the full mass of a wing including the blade and the setae is 0.0235 µg for PP2. This constitutes 0.97% of the mass of the body. The isometrically rescaled values for PP4, PP5 and PP12 are given in Supplementary Table 2.

Subsequently, we calculated the moments of inertia with respect to the hinge point which is the centre of solid body rotation of the wing model used in the aerodynamic simulations. We neglected the out-of-plane deviation of the blade, calculated its surface density as mass divided by planar projection area and applied a simple rectangular 2D quadrature rule with a discretization step of 50 µm. The moments of inertia of the individual setae were calculated using the formula for a thin rod at an angle and the parallel axis theorem. The summation of the above contributions yielded the following values for the non-zero components of the bristled wing inertia tensor as shown in Supplementary Table 2. Note that the direction of the x-axis is from the trailing edge to the leading edge, y-axis is from the proximal part to the apex, the z-axis is complimentary and the origin of the coordinate system is at the wing hinge (Supplementary Fig. 3).

Supplementary Table 2. Inertia properties of the wings and elytron.

Ind. #	*R* (mm)	Body part	h_m (µm)	*m* (µg)	J_{xx} (µg·µm^2)	J_{yy} (µg·µm^2)	J_{zz} (µg·µm^2)	J_{xy} (µg·µm^2)
PP2	0.493	bristled wing	–	0.024	1450	120	1570	−160
		bristled wing, cylindrical setae	–	0.042	3370	360	3730	−370
		membranous wing	0.73	0.126	11900	1900	13800	−1440
		membranous wing	0.85	0.144	13700	2210	15900	−1660
		membranous wing	0.98	0.164	15700	2550	18200	−1900
		membranous wing	1.12	0.185	17800	2910	20700	−2160
		elytron	–	0.174	7160	300	7460	820
PP4	0.505	bristled wing	–	0.025	1640	135	1770	−180
		elytron	–	0.187	8080	338	8410	925
PP5	0.495	bristled wing	–	0.024	1480	122	1600	−163
		elytron	–	0.176	7310	306	7610	837
PP12	0.485	bristled wing	–	0.022	1340	111	1450	−147
		elytron	–	0.166	6600	276	6870	756

We used the same method to evaluate the mass and the moments of inertia of a hypothetical wing with smooth cylindrical setae having an equivalent circular cylinder section (i.e., having a larger diameter and producing the same aerodynamic force as the setae with secondary outgrowths). This diameter, b = 1.9 µm, is 2.1 times as large as the stem diameter of a real seta[14]. The full mass of a wing including the blade and the aerodynamically equivalent cylindrical setae is 0.0421 µg. The corresponding components of the inertia tensor are about twice as large as for the original wing (Supplementary Table 2). The mass and the moment of inertia of the equivalent membranous wing have been determined for three different assumed values of membrane thickness, h_m = 0.73, 0.85 and 1.12 µm, corresponding to the membranes of *Trichogramma telengai* (Hymenoptera: Trichogrammatidae, l_b = 0.45 mm), *Orthoperus atomus* (Coleoptera: Corylophidae, l_b = 0.8 mm) and *Limnebius atomus* (Coleoptera: Hydraenidae, l_b = 1.1 mm), respectively. The membrane thickness of 0.98 µm is the minimum thickness of the membranous part (blade) of a *P. placentis* wing. Membrane thickness values of the wings were obtained by measuring histological sections 0.5 µm thick made on a Leica microtome with the diamond knife, after the fixation in araldite. The measurements were performed on an Olympus BX43 microscope. We used the above-mentioned 2D quadrature rule to calculate the area enclosed by the outer contour of the blade and the line connecting the tips of the setae[14], as well as the moments of area. After multiplying by the membrane thickness and the cuticle density, we found the equivalent membranous wing mass values and moments of inertia shown in Supplementary Table 2.

To evaluate the mass and the moments of inertia of the elytron, we used the same method as for the central membranous part of the wing. The mass was estimated using a 3D model as 0.1740 µg. Then, the moments of inertia were obtained by 2D integration, neglecting the out-of-plane deviation and assuming uniform thickness distribution, yielding the values given in Supplementary Table 2.

Section 10 – Kinematic model for CFD

The wings and the elytra rotate about the hinge points fixed in the body reference frame (Supplementary Fig. 6). For the CFD simulation, the angles ϕ, θ and ψ originally measured in the flight experiment are converted into the Euler angles compatible with the CFD solver[16], relative to the anatomical stroke plane. We first calculated the Euler angles in the same reference frame of the aerodynamic stroke plane as used in the definition of ϕ, θ and ψ: the positional angle is equal to ϕ, the elevation angle is equal to θ, but the feathering angle is defined as the rotation angle about the longitudinal axis of the wing, i.e., it is different from ψ. Denoting the feathering angle as α, and the respective angle between the

wing plane and the stroke plane as $\psi^{\dagger}(\alpha)$, we used a genetic optimization algorithm CMA-ES[17] to find the values of α that minimize the residue $|\psi^{\dagger}(\alpha) - \psi|$ and thus define the same wing orientation as the originally calculated angle ψ. We calculated the phase average and left-right average values of α as described above.

We then used the morphological model of the wing to define marker points. Each seta contains 100 points uniformly distributed along the axis between its root and its tip. A total of 600 points are placed along the blade midline. Knowing the Euler angles of the wings in the aerodynamic stroke plane reference frame and the distance between the hinge points, it is possible to determine the spatial position of the markers on the flapping wings. We stored those instantaneous positions at 201 time instants between t = 0 and t = T. Subsequently, we evaluated the instantaneous orientation of the body at the same time instants using the mean value and the first Fourier mode of the body angle χ. We defined the anatomical stroke plane as the plane that moves with the body and coincides with the aerodynamic stroke plane when the body angle is equal to its mean value.

The CFD simulation requires the Euler angles of the wing measured with respect to the anatomical stroke plane. Their values are found using optimization. We started from a random guess, evaluated the corresponding position of the markers on the wings in 3D, calculated the root-mean-square distance between these markers and those determined earlier in the aerodynamic stroke plane reference frame, and employed the CMA-ES to minimize that distance. The optimization was performed for the wings and for the elytra. It yielded the values of positional, elevation and feathering angles with respect to the anatomical stroke plane that oscillates with the body. The values are available at 201 time instants between t = 0 and t = T. To evaluate the Euler angles at any arbitrary time instant and ensure periodicity, we spline-interpolated on a uniform grid with 1024 points, applied the fast Fourier transform, and stored the first M = 11 modes in the form of the sine and cosine coefficients, as required by the CFD solver[16].

The orientation of the body was prescribed by defining the time evolution of the pitch angle between the horizontal plane in the laboratory reference frame and the longitudinal axis of the body x_b. Note that in this study the body pitch angle is positive when the body is oriented nose up (this is opposite to the sign convention used previously[16]). Fourier analysis was performed and only the mean, the first sine and the first cosine coefficients were retained.

The anatomical stroke plane does not move with respect to the body. It is tilted by an angle η relative to the body transverse plane y_b-z_b, in the notation of Engels et al (2015)[16]. so that the average orientation of the anatomical stroke plane coincides with the orientation of the original aerodynamic stroke plane[16].

Extended Data Fig. 2c summarizes the time evolution of the Euler angles of the wing and elytron relative to the body, and the body pitch angle, for four individuals, as used in the CFD simulations. The anatomical stroke plane angle η takes the values −33.5, −36.6, −28.5 and −35.8° for the four analyzed individuals, respectively.

Section 11 – CFD simulation setup

The computational fluid dynamics analysis was performed using an open-source Navier-Stokes solver WABBIT[18]. It was based on the artificial compressibility method to enforce the velocity-pressure coupling, volume penalization method to model the no-slip condition at the solid surfaces, and dynamic grid adaptation using the wavelet coefficients as refinement indicators. The morphology and kinematics described above define the time-varying position of the volume penalization mask function and the internal velocity field of the solid parts.

The computational domain is a $12R \times 12R \times 12R$ cube (Supplementary Fig. 7), where R is the wing length. The volume penalization is used in combination with periodic external boundary conditions to enforce the desired far-field velocity[18]. The computational domain is decomposed in nested Cartesian blocks, each containing $25 \times 25 \times 25$ grid points. The blocks are created, removed and redistributed among parallel computation processes so as to ensure the maximum refinement level near the solid boundaries and constant wavelet coefficient thresholding otherwise during the simulations (Supplementary Fig. 8). Since the flow is far from being turbulent but Δx_{min} is small for geometrical reasons, the threshold value for thresholding wavelet coefficients is fixed at $\varepsilon = 10^{-2}$. With this choice, the most refined grid blocks are those that contain the solid surface.

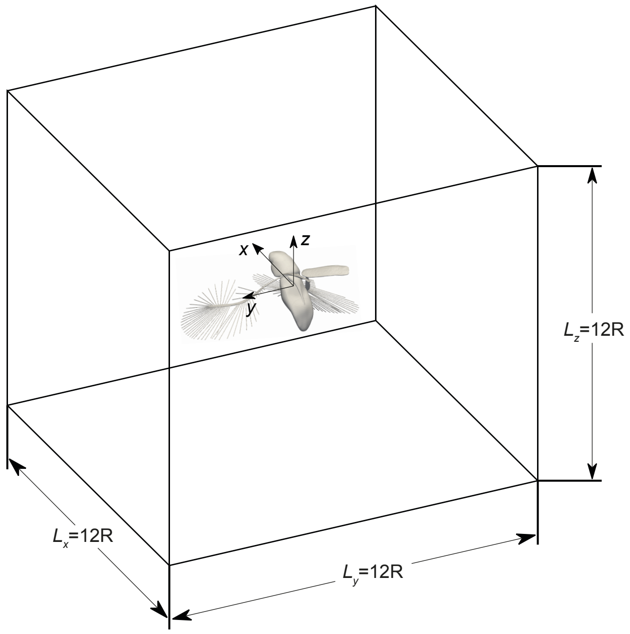

Supplementary Figure 7. Computational domain.

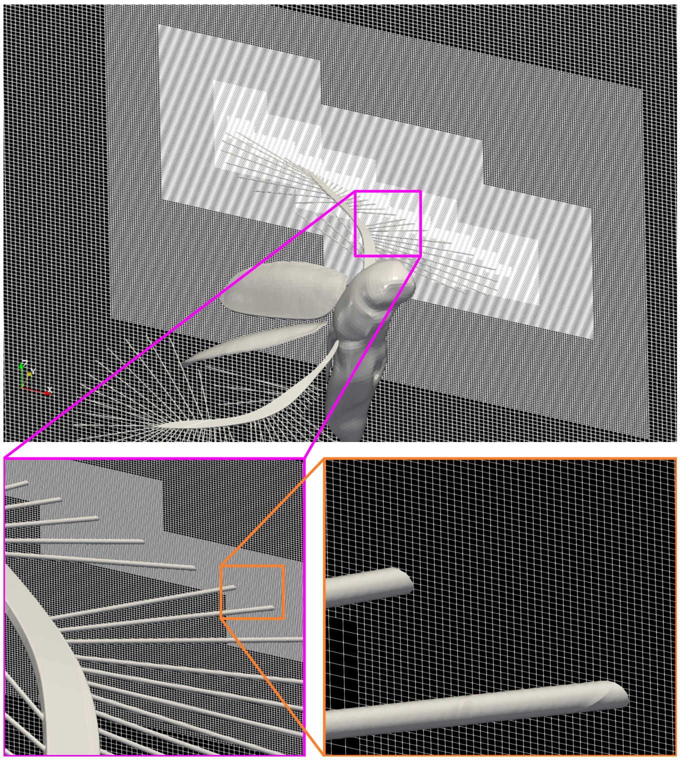

Supplementary Figure 8. Visualization of the block-based adaptive grid used in the simulations. The grid is refined on the surface of the insect, including the setae, in order to resolve all features of the flow.

The numerical simulations started from the quiescent air condition, continued for a time period of two wingbeat cycles with a coarse spatial grid resolution of $\Delta x_{min} = 0.00781R$ to let the flow develop to its ultimate periodic state, then the spatial discretization size was allowed to reduce to $\Delta x_{min} = 0.00098R$ if the wing was bristled or to $\Delta x_{min} = 0.00195R$ if it was membranous, and the simulation continued for one more wing beat period

to obtain high-resolution results. The air temperature was 25 °C in all cases, air density ρ = 1.197 kg/m^3, and the kinematic viscosity of the air $\nu = 1.54 \cdot 10^{-5}$ m^2/s.

All numerical simulations were performed with the artificial speed of sound prescribed as $c_0 = 30.38fR$. This value had been selected based on the preparatory comparison between CFD simulations and dynamically scaled rotating wing model experiments[14]. The values assigned to the volume penalization parameter varied in proportion with $\Delta x_{min}^2/\nu$. The high-resolution results were obtained with $C_\eta = 6.48 \cdot 10^{-7}f$, $7.50 \cdot 10^{-7}f$, $7.29 \cdot 10^{-7}f$ or $6.66 \cdot 10^{-7}f$, depending on the individual number PP2, PP4, PP5 or PP12, respectively.

The low Reynolds number of the flow requires that the step of explicit time integration be proportional to Δx_{min}^2. This constraint is handled by using the Runge–Kutta–Chebyshev time-stepping schemes[19] with large stability regions designed so as to encompass all eigenvalues of the linear part of the differential operator in each of the four cases. These schemes are second-order accurate, and the number of stages s is relatively large to guarantee the desired stability: s = 22, 20, 21 or 22 for the individuals PP2, PP4, PP5 or PP12, respectively.

Section 12 – Details of the flow around the setae

During the dorsal recovery stroke, the wings are brought closely together (Extended Data Fig. 3, t = 2.0). The flow velocity field is dominated by the downwash of the preceding power stroke. As the wings approach, they eject the air between them which results in an increased pressure. In the following power stroke (Extended Data Fig. 3, t = 2.37), the wings move downward at a high angle of attack. Even though the gap distance between the setae is rather large, the wings are seen to behave as if they were solid, with little fluid leaking between the setae. The pressure on the bottom surface is increased, while it decreases on the top surface of the wing. The flow exhibits the typical features of flapping flight, even though the leading edge vortex is, due to the elevated viscosity, much weaker compared to larger animals. The ventral recovery stroke (Extended Data Fig. 3, t = 2.60) is similar to the dorsal one. We show the moment when the wings start to open, which results in an increased pressure on the outside. Finally, during the second power stroke (Extended Data Fig. 3, t = 2.82), the flow is again similar to the first power stroke.

At all time instances, small, localized flow features are observed in the direct vicinity of the bristles, along with the more global flow features.

Section 13 – CFD results

The time evolution of the aerodynamic force acting on the beetle is shown in Extended Data Fig. 4a. In all cases, the vertical force has two positive peaks during the power strokes, and it is negative during the recovery strokes. The horizontal force also peaks during the two power strokes, but the sign changes from negative to positive. The mean vertical force is positive. The calculated value is large enough to support the weight of the insect, with the small discrepancy which is partly due to the non-zero vertical acceleration of the beetle in the experiment, and partly due to modelling error. The mean horizontal force is small. These mean values are shown in Supplementary Table 3.

Newton’s second law provides a useful relation for diagnosing the accuracy of the aerodynamic force computation. Supplementary Table 3 shows the two components of the residual error in the law of motion evaluated after substituting the computed mean force and the measured acceleration,

$$e_x = (\bar{F}_{ax} - m\bar{a}_x)/mg \cdot 100\%, \quad (2)$$

$$e_z = (\bar{F}_{az} - m(\bar{a}_z + g))/mg \cdot 100\%. \quad (3)$$

The r.m.s. values of e_x and e_z across the four individuals are, respectively, 9.4% and 12.1%. Note that this error includes the inaccuracy of the CFD modelling and the uncertainty of the body mass evaluation.

The contribution of the wings, the elytra and the body to the total vertical aerodynamic force is shown in Extended Data Fig. 4b. The main contribution is always from the wings. The elytra produce a noticeable positive vertical force towards the end of the morphological downstroke, but this force cancels out with the negative force generated later.

The lift-drag decomposition of the vertical aerodynamic drag shows substantial inter-individual variability (Extended Data Fig. 4c). The instantaneous force is always dominated by the drag, but its large positive and negative contributions cancel each other out after time averaging. Instead, the vertical aerodynamic force due to lift is for most of the time positive. Therefore, the mean vertical aerodynamic force is up to 68%, 84%, 79% and 40% due to the lift and 32%, 16%, 21% and 60% due to the drag for the four beetles, respectively.

Supplementary Table 3. Input and output parameters of the CFD simulations specific to individuals.

Individual number	PP2	PP4	PP5	PP12
Flapping frequency f (Hz)	171.0	190.9	190.9	183.6
Anatomical positional angle amplitude Φ (°)	182.9	174.8	182.7	170.9
Aerodynamical stroke plane angle β (°)	8.2	12.6	2	9.7
Anatomical stroke plane angle χ (°)	−33.5	−36.6	−28.5	−35.8
In-flight body length l_b (mm)	0.395	0.405	0.397	0.389
Wing length R (mm)	0.493	0.505	0.495	0.485
Mean velocity at radius or gyration R_g (m/s)	0.444	0.475	0.480	0.438
Estimated body mass m_b (μg)	2.43	2.62	2.46	2.32
Mean horizontal velocity $\bar{V}_x$ (m/s)	0.052	0.050	0.046	0.077
Mean vertical velocity $\bar{V}_z$ (m/s)	0.042	0.062	0.057	−0.004
Mean horizontal acceleration $\bar{a}_x$ (m/s^2)	−0.695	−0.097	0.089	−1.178
Mean vertical acceleration $\bar{a}_z$ (m/s^2)	1.023	0.130	−0.052	4.295
Mean horizontal aerodynamic force $\bar{F}_{ax}$ (μgf)	−0.42	−0.27	0.04	−0.57
Mean vertical aerodynamic force $\bar{F}_{az}$ (μgf)	2.71	2.38	2.98	3.26
Horizontal residual error e_x	−10.4%	−9.5%	0.8%	−12.6%
Vertical residual error e_z	0.9%	−10.5%	21.5%	−3.3%
Mean body mass specific power $\bar{P}$ (W/kg)	27.6	29.5	34.0	24.4
Peak body mass specific power P_{max} (W/kg)	109.0	115.4	164.2	90.4
Pitching amplitude $\Delta\chi$ (°) measured	29.4	24.7	28.0	32.7
Pitching amplitude $\Delta\chi$ (°) computed with elytra	36.2	28.2	27.8	30.2
Pitching amplitude $\Delta\chi$ (°) computed w/o elytra	69.8	75.3	76.6	76.0

The wing tip trajectories shown in Supplementary Fig. 9, coloured according to the sign of the vertical aerodynamic force of the wings, emphasize that the wing beat cycle consists of two power strokes and two recovery strokes. During the power strokes, the wings produce a large aerodynamic force that has an upward component (e.g., at t/T = 0.29 and 0.82). The wing velocity at the same time is downward, because it is tangential to the trajectory and the wing elevation angle decreases during the power stroke. Therefore, large portion of the aerodynamic force can be explained by the drag. However, at t/T = 0.29 for example, the vector diagrams show that the force is not perfectly anti-aligned with the velocity. It is deflected upwards. This deflection can be explained by the aerodynamic lift which is perpendicular to the direction of wing motion. This positive deflection is present even during the recovery stroke, e.g., at t/T = 0.19, for PP2, PP5 and PP12. when the aerodynamic force is downward. During the recovery strokes, the wings clap or near-clap and move upwards. This motion generates a downward aerodynamic force. The recovery motion is slow; therefore the aerodynamic force is small in magnitude, but it acts for a longer time. Hence, the net time-average vertical aerodynamic force is an order of magnitude smaller than the peak at the power stroke.

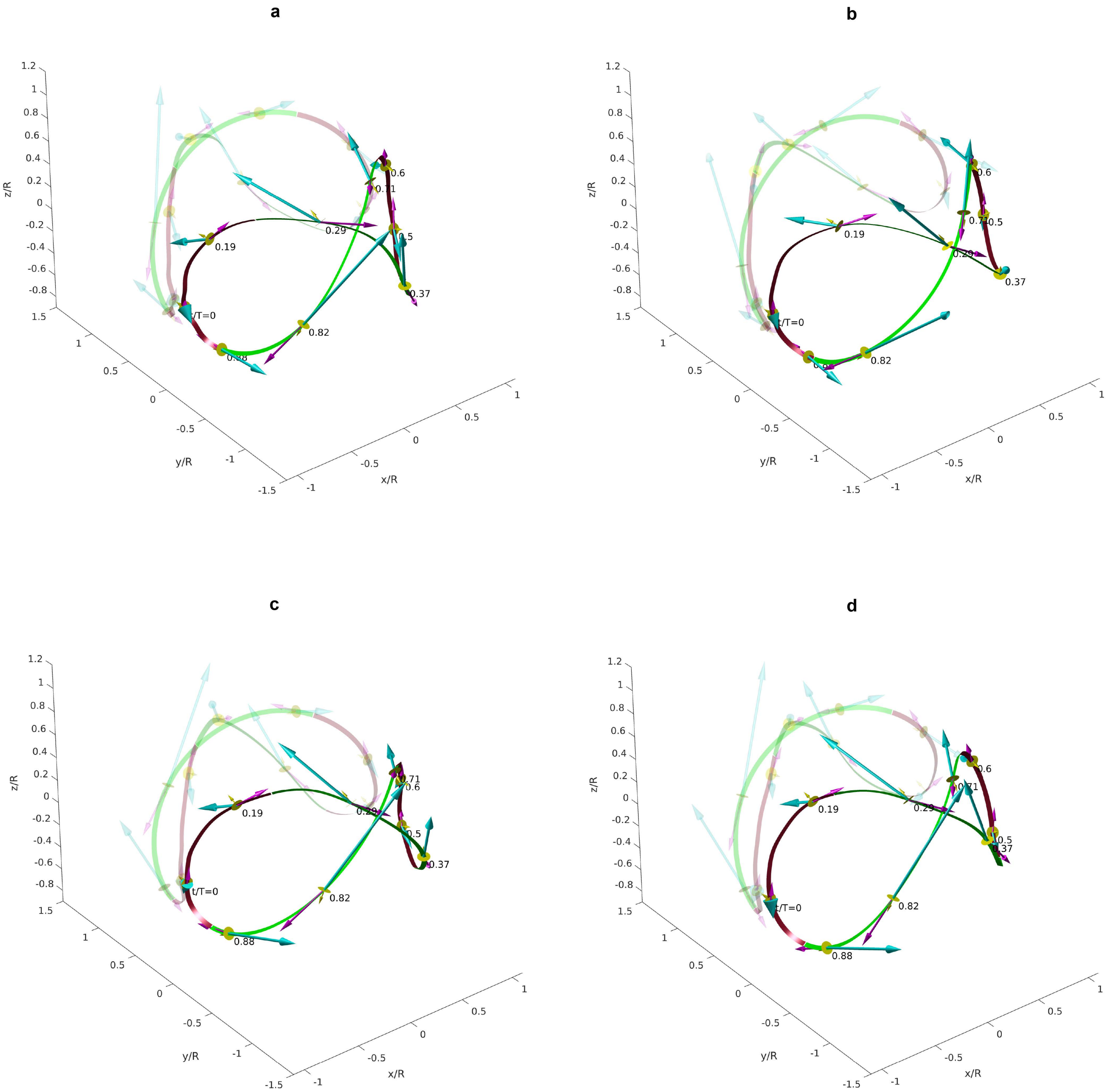

Supplementary Figure 9. 3D reconstruction of the wing-tip trajectories (continuous lines), aerodynamic force vectors (cyan arrows), velocity vectors (magenta arrows) and wing orientation (yellow circles and arrows). Click to view interactive 3D models**. a,** PP2. **b,** PP4. **c,** PP5. **d,** PP12.

The body-mass-specific mechanical power shown in Extended Data Fig. 6 in all cases peaks during the power strokes. The aerodynamic contribution is dominant. The largest peak is for PP5 (164.2 W/kg) and the smallest peak is for PP12 (90.4 W/kg). The mechanical power essentially remains positive through the entire wingbeat cycle period, although occasionally it takes small negative values. For example, its minimum value for PP2 is −5.5 W/kg at t/T = 0.9 and for PP5 it is −8.3 W/kg at t/T = 0.52. Therefore, this type of wing beat motion does not require any substantial elastic energy storage. The mean body-mass-specific mechanical power is equal to 27.6, 29.5, 34.0 and 24.4 for PP2, PP4, PP5 and PP12, respectively.

The aerodynamic pitching moment that the wings exert on the body (Extended Data Fig. 7) is consistently negative (nose-up) during the first recovery and power strokes (approximately, morphological downstroke). It becomes positive (nose-down) during the second recovery and power strokes (approximately, morphological upstroke). The inertial pitching moment due to the elytra changes from positive to negative at about $t/T = 0.4$ when the elytra begin to close to compensate for that changing direction of the aerodynamic pitching moment. The aerodynamic pitching moment of the elytra and the inertial pitching moment of the wings are small.

Section 14 – Mechanical power calculation

The mechanical power expenditure was evaluated using a previously described method of computational fluid dynamics[16]. Let us recapitulate it. The mathematical model approximates the wings, elytra and body at rigid solid objects connected by spherical joints. The internal structure of the animal is not modelled: instead, the skeletal and muscle action is substituted, regardless of its physiological type, by actuation torques

$$\vec{M}_{actu_j}, \tag{4}$$

where the subscript j stands for the joint number and takes the values of 1, 2, 3 and 4 for the left wing, right wing, left elytron and right elytron, respectively. The mechanical power expended on the actuation of the j-th joint is equal to

$$P_j = \vec{\Omega}_j \cdot \vec{M}_{actu_j}, \tag{5}$$

where $\vec{\Omega}_j$ is the angular velocity of the j-th part relative to the body. The total mechanical power P of the insect is obtained by summation. The actuation torque applied on the j-th part at the hinge is balanced by the aerodynamic and the inertia torques about the hinge point,

$$\vec{M}_{actu_j} = -\left(\vec{M}_{aero_j} + \vec{M}_{inert_j}\right). \tag{6}$$

This suggests the following decomposition of the total mechanical power into the aerodynamic and the inertial components,

$$P = P_{aero} + P_{inert}, \tag{7}$$

where

$$P_{aero} = -\sum_{j=1}^{4} \vec{\Omega}_j \cdot \vec{M}_{aero_j},\ P_{inert} = -\sum_{j=1}^{4} \vec{\Omega}_j \cdot \vec{M}_{inert_j}. \tag{8}$$

Calculation of the aerodynamic torques is implemented as volume integration of the penalization term in the penalized momentum equation in the fluid solver[16]. This is equivalent to the more commonly used surface integration of fluid stresses.

The inertial torque is equal to

$$\vec{M}_{inert_j} = -\left(\mathrm{J}_j \cdot \dot{\vec{\Omega}}_j + \vec{\Omega}_j \times \mathrm{J}_j \vec{\Omega}_j\right), \tag{9}$$

where J_j is the tensor of inertia. It is convenient to evaluate the above formula in the local coordinate frames of the respective wing/elytron, see Supplementary Figs. 3, 4, 5 and Extended Data Fig. 2a. The tensor of inertia is symmetric and, in the thin plate approximation, it contains only four non-zero entries: J_{xx}, J_{yy}, J_{zz} and J_{xy}. They were calculated as described in the dedicated section of this document.

Section 15 – The effect of elytra on body pitch oscillation

To estimate the effect of elytra on the body pitch oscillation, let us consider the angular momentum conservation about the body pitch axis,

$$J_b \frac{d^2(-\chi)}{dt^2} = M, \tag{10}$$

where $M = M_{ba} + M_{wi} + M_{wa} + M_{ei} + M_{ea}$, M_{ba} is the body aerodynamic pitching moment, M_{wi}, M_{wa} are the inertial and aerodynamic components of the pitching moment due to the flapping wings and M_{ei}, M_{ea} are the inertial and aerodynamic components of the pitching moment due to the elytra, respectively. Note that the "–" sign appears in (10) because the positive direction of χ is nose-up (we follow the aeronautical convention) but the positive pitching moment direction is nose-down to be consisted with the right-handed coordinates used in the analysis. Restricting our attention to time-periodic solutions of (10) with the period T, we solve it using the Fourier transform,

$$J_b k^2 \hat{\chi} = \hat{M}, \tag{11}$$

where the hat denotes the Fourier transform of the temporal evolution of a quantity. In the practical calculation, we use the fast Fourier transform in MATLAB (MathWorks, U.S.A.). The time evolution $M(t)$ is sampled on a uniform grid containing $N = 1024$ points, k takes integer values from $-N/2+1$ to $N/2$, and we find

$$\hat{\chi}(k) = \begin{cases} \hat{M}(k)/J_b k^2 & if\ k \neq 0\ and\ k \neq N/2, \\ 0 & if\ k = 0\ or\ k = N/2. \end{cases} \tag{12}$$

Then, the inverse fast Fourier transform is applied to calculate $\chi(t)$ samples on the same uniform grid of points between 0 and T. We mainly want to know the amplitude $\Delta\chi = \max(\chi) - \min(\chi)$.

To avoid additional CFD simulation that would require full fluid-solid coupling, we neglect the dependency of M_{ba}, M_{wa} and M_{ia} on the difference between $\chi(t)$ prescribed in the original CFD simulation and that computed by solving (10). This is justified because the amplitude of the wing motion is much greater than the amplitude of the body motion, and the body and the elytron aerodynamic pitching moments are negligible. The values of $\Delta\chi$ obtained from (10) with M_{ba}, M_{wi}, M_{wa}, M_{ei} and M_{ea} taken from the CFD simulations agree well with $\Delta\chi$ measured in the experiment, see Supplementary Table 3. This additionally validates the CFD model.

Extended Data Fig. 7 further demonstrates that M_{ea} is negligible. But the inertial pitching moment due to the elytra M_{ei} is essential. If we fully neglect it, substituting $M = M_{ba} + M_{wi} + M_{wa}$ into (10), we find values of $\Delta\chi$ that are 1.9 to 2.7 times larger than in the previous cases with the elytra. The maximum body pitching angle $\max(\chi)$ becomes greater than 90°. We therefore conclude that the elytra act as inertial brakes that prevent overturning.

Section 16 – Numerical convergence

Additional numerical simulations have been carried out to evaluate the accuracy of the CFD simulations. In addition to the original simulation of PP2 with the maximum number of refinement levels $j_{max} = 9$, we have repeated similar computations with $j_{max} = 8$ and $j_{max} = 7$. These new runs have a coarser grid resolution in the neighbourhood of the solid boundaries (see the values of $\Delta x_{min}/R$ in Supplementary Table 4).

Supplementary Table 4. Numerical convergence data.

j_{max}	7	8	9
$\Delta x_{min}/R$	0.00391	0.00195	0.00098
Mean horizontal aerodynamic force $\bar{F}_{ax}$ (μgf)	−0.4551	−0.4357	−0.4238
Mean vertical aerodynamic force $\bar{F}_{az}$ (μgf)	2.1292	2.5077	2.7066
Mean body mass specific power $\bar{P}$ (W/kg)	24.4738	26.5411	27.5108

Extended Data Fig. 8 shows the time variation of the aerodynamic force components and the body mass specific aerodynamic power in these three computations. All three show similar qualitative trends. The results for $j_{max} = 8$ and $j_{max} = 9$ are close enough for making quantitative conclusions. Thus, the difference between the mean vertical force computed with $j_{max} = 8$ and with $j_{max} = 9$ is less than 9% of the body

weight. The horizontal force difference is less than 1% of the body weight. The mean aerodynamic power differs by less than 4%.

Section 17 – On the fluid-dynamic added mass effect

It is common to use the concept of added mass to describe the acceleration reaction of a solid body moving in a fluid. An order-of-magnitude estimate can be obtained by the following formula[20]:

$$M = \frac{\rho D^3}{3}, \text{ where } D = 2(S/\pi)^{3/2}, \quad (13)$$

M is the added mass, ρ is the air density (1.197 kg/m^3) and S is the wing area. Assuming the added air mass of the bristled wing (for its low leakiness) is about the same as the added air mass of the membranous wing model, we find M = 0.0233 μg, which is close to the mass of the bristled wing. We do not elaborate further on this question, because the acceleration reaction at $Re \approx 10$ strongly depends on the flow velocity, which undermines the practicality of the added mass concept.

Section 18 – Comparison with larger beetles and tiny insects of other orders

The large 85° amplitude of the wing's deviation angle (Fig. 2b, d) is in striking contrast to most larger beetles (e.g., 9° in *Trypoxylus dichotomus* (Linnaeus, 1771)[21] and 16° in *Batocera rufomaculata* (De Geer, 1775)[22]). Small stroke deviation angles are typical of lift-based hovering flight during which wings move within a stroke plane perpendicular to the direction of the net aerodynamic force. Large deviation angles, by contrast, are typical for drag-based flight and have been observed in different families of small wasps, midges and thrips[23,24]. Nevertheless, the fast power strokes and slow recovery strokes found in *P. placentis* are unique. The time course of vertical aerodynamic force with two narrow positive peaks and two shallow negative valleys has more in common with smaller representatives of other families[23,24] than with larger beetles[21] (Extended Data Fig. 9a). This also includes the elevated instantaneous reaction loads exerted on the skeletomuscular system. Our calculations suggest that ptiloptery is an efficient solution to the problem of reducing the inertial component of those peak loads. Ptiloptery thus allows *P. placentis* to increase wing size in order to compensate for gravity without increasing inertial costs during wing flapping. This might explain why pliliids tend to have larger wings relative to their body length than other Staphylinoidea[1,25,26,27] (Extended Data Fig. 9b, two-samples Wilcoxon test p = 0.000143).

Let us elaborate on the comparison with *E. formosa* Gahan, 1924 [24]. It has the wing length R = 0.61 mm, wing area S = 0.14 mm^2, flapping frequency f = 361 Hz and amplitude Φ = 144° (the data corresponds to individual EF1[24]). Its body mass can be estimated as m_b = 20 μg, considering weight support in hovering flight[24]. In comparison with *P. placentis* individual PP2, the body mass is 8 times as large (which suggest that the body is probably about twice as long), but the wing is only 24% longer. The flapping amplitude is 20% less, but the flapping frequency is twice as high.

The aerodynamic power has been computed in the reference[24]. The inertial power can be estimated using the same method as for *P. placentis*. To this end, we used the available wing kinematics[24] in terms of Euler angles, and a rough estimate of the wing inertia tensor. We estimated the wing mass using the allometric trend (SI 3) as $m_w = 2.17 S_{\mathrm{mm}}^{1.42}$ = 0.13 μg, where S_{mm} is the wing area in mm^2. Moments of inertia can be estimated using a functional relation $J = \text{const} \cdot m_w R^2$ known from the previous work on Diptera[7], but with the dimensionless constant fitted to Hymenoptera data[10]. We obtain J_{xx} = 9390 μg·μm^2, J_{yy} = 308 μg·μm^2, J_{zz} = 9700 μg·μm^2, J_{xy} = −219 μg·μm^2. This estimate assumes similarity with the spanwise and chordwise dimensionless mass distribution of *E. formosa* and *B. ingitus*. This is plausible, since most of the mass in an *E. formosa* wing belongs to the relatively large membranous part and the leading-edge vein. The long setae in the proximal part make little contribution, and in the distal part the setae are short.

The results for the body mass specific mechanical power as a function of time are shown in Extended Data Fig. 10a. During power strokes, *E. formosa* produces peaks of the inertial power that are 50% as large as the concomitant peaks of the aerodynamic power. Consequently, the peak total power is greater for *E. formosa* than for *P. placentis* by 50%, although the mean body mass specific mechanical power expenditure is nominally equal in the two individuals of the two species (24 W/kg in *E. formosa*, assuming perfect elastic storage, vs 28 W/kg in *P. placentis*) (Extended Data Fig. 10b).

The volume of the flight muscles of five specimens was measured on confocal stacks in Bitplane Imaris. Samples were prepared in the same way as for measuring body volume, with additional staining with 0.05% Rhodamin B buffer solution after depigmentation for 7 days at 4 °C. The total volume of the muscles involved in the raising and lowering of the wing[28] is 0.158 ± 0.003 nL^3 (5.6% of the body volume). Considering the muscle density value as 1060 kg/m^3, the total flight muscles mass is 6.9% of the body mass. Based on aerodynamic power and flight muscle mass, the mass-specific power of *P. placentis* muscles is 350 W/kg at a frequency of 171 Hz, which coincides with the specific power output of asynchronous muscles predicted by Pennycuick & Rezende and adopted by Ellington[29].

Section 19 – Ptiloptery and the original flying style help beetles to fly faster

Let us put together four important observations.

(I) We have found that bristled wings are lighter than the equivalent membranous wings and have smaller moments of inertia.

(II) The inertial forces are proportional to acceleration, i.e., to flapping frequency squared. The inertial power therefore varies as frequency cubed.

(III) Since the Reynolds number is relatively low, the aerodynamic force of a wing is proportional to its velocity raised to some exponent $\gamma < 2$. The velocity is proportional to the flapping frequency. The instantaneous aerodynamic power, defined as a product of the force and velocity, is proportional to the frequency raised to the exponent of $1 + \gamma < 3$.

(IV) The wings clap dorsally and ventrally even at low speeds. At high speeds, unable to further increase the flapping amplitude, the beetle unavoidably increases the flapping frequency to produce enough aerodynamic thrust to counter the body drag, keeping the flapping amplitude constant. Consequently, the peaks of the inertial power increase, and they do so faster than the aerodynamic power. However, the flapping frequency cannot be increased beyond the point when the muscles become unable to sufficiently accelerate the wing upon power stroke, even if the mean aerodynamic power is low.

We arrive at the following conclusion: for a beetle as miniature as *P. placentis*, with the cycle-averaged Re as low as 9, low wing moment of inertia is a prerequisite of fast flight. This is achieved by ptiloptery.

References

1. Polilov, A. A., Reshetnikova, N. I., Petrov, P. N. & Farisenkov, S. E. Wing morphology in featherwing beetles (Coleoptera: Ptiliidae): Features associated with miniaturization and functional scaling analysis. *Arthropod Struct. Dev*. **48**, 56–70 (2019).

2. Ellington, C. P. The aerodynamics of hovering insect flight. II. Morphological parameters. *Philos. Trans. R. Soc. London. B, Biol. Sci*. **305**, 17–40 (1984).

3. Ennos, A. R. The inertial cause of wing rotation in Diptera. *J. Exp. Biol*. **140**, 161–169 (1988).

4. Ennos, A. R. Inertial and aerodynamic torques on the wings of Diptera in flight. *J. Exp. Biol*. **142**, 87–95 (1989).

5. Ha, N. S., Truong, Q. T., Goo, N. S. & Park, H. C. Relationship between wingbeat frequency and resonant frequency of the wing in insects. *Bioinspir. Biomim*. **8**, 046008 (2013).

6. Chen, M. W., Zhang, Y. L. & Sun, M. Wing and body motion and aerodynamic and leg forces during take-off in droneflies. *J. R. Soc. Interface* **10**, 20130808 (2013).

7. Hargrove, J. W. The flight performance of tsetse flies. *J. Insect Physiol*. **21**, 1385–1395 (1975).

8. Lehmann, F. O. & Heymann, N. Dynamics of in vivo power output and efficiency of *Nasonia* asynchronous flight muscle. *J. Biotechnol*. **124**, 93–107 (2006).

9. Bergou, A. J., Ristroph, L., Guckenheimer, J., Cohen, I. & Wang, Z. J. Fruit flies modulate passive wing pitching to generate in-flight turns. *Phys. Rev. Lett*. **104**, 148101 (2010).

10. Kolomenskiy, D. et al. The dynamics of passive feathering rotation in hovering flight of bumblebees. *J. Fluids Struct*. **91**, 102628 (2019).

11. Farisenkov, S. E., Lapina, N. A., Petrov, P. N. & Polilov, A. A. Extraordinary flight performance of the smallest beetles. *Proc. Natl. Acad. Sci. U. S. A.* **117**, 24643–24645 (2020).

12. Wan, K. Y. & Goldstein, R. E. Coordinated beating of algal flagella is mediated by basal coupling. *Proc. Natl. Acad. Sci. U. S. A.* **113**, E2784–E2793 (2016).

13. Ellington, C. P. The aerodynamics of hovering insect flight. VI. Lift and power requirements. *Philos. Trans. R. Soc. London. B, Biol. Sci*. **305**, 145–181 (1984).

14. Kolomenskiy, D. et al. Aerodynamic performance of a bristled wing of a very small insect: Dynamically scaled model experiments and computational fluid dynamics simulations using a revolving wing model. *Exp. Fluids* **61**, (2020).

15. Vincent, J. F. V. & Wegst, U. G. K. Design and mechanical properties of insect cuticle. *Arthropod Struct. Dev*. **33**, 187–199 (2004).

16. Engels, T., Kolomenskiy, D., Schneider, K. & Sesterhenn, J. FluSI: A novel parallel simulation tool for flapping insect flight using a Fourier method with volume penalization. *SIAM J. Sci. Comput*. **38**, S3–S24 (2015).

17. Hansen, N., Müller, S. D. & Koumoutsakos, P. Reducing the time complexity of the derandomized evolution strategy with covariance matrix adaptation (CMA-ES). *Evol. Comput*. **11**, 1–18 (2003).

18. Engels, T., Schneider, K., Reiss, J. & Farge, M. A wavelet-adaptive method for multiscale simulation of turbulent flows in flying insects. *Commun. Comput. Phys.* **30**, 1118-1149 (2021).

19. Verwer, J. G., Sommeijer, B. P. & Hundsdorfer, W. RKC time-stepping for advection-diffusion-reaction problems. *J. Comput. Phys*. **201**, 61–79 (2004).

20. Brown, A., Thomson, J. & Rusch, C. Hydrodynamic coefficients of heave plates, with application to wave energy conversion. *IEEE J. Ocean. Eng*. **43**, 983–996 (2018).

21. Oh, S., Lee, B., Park, H., Choi, H. & Kim, S. T. A numerical and theoretical study of the aerodynamic performance of a hovering rhinoceros beetle (*Trypoxylus dichotomus*). *J. Fluid Mech.* **885**, A18 (2019).

22. Urca, T., Debnath, A. K., Stefanini, J., Gurka, R. & Ribak, G. The Aerodynamics and power requirements of forward flapping flight in the mango stem borer Beetle (*Batocera rufomaculata*). *Integr. Org. Biol*. **2**, obaa026 (2020).

23. Cheng, X. & Sun, M. Very small insects use novel wing flapping and drag principle to generate the weight-supporting vertical force. *J. Fluid Mech*. **855**, 646–670 (2018).

24. Lyu, Y. Z., Zhu, H. J. & Sun, M. Flapping-mode changes and aerodynamic mechanisms in miniature insects. *Phys. Rev. E* **99**, 1–12 (2019).

25. Oertli, J. Interspecific scaling relative size change of wing beat frequency and morphometrics in flying beetles Coleoptera. *Mitt. Schweiz. entomol. Ges*. **64**, 139–154 (1991).

26. Gibb, H. et al. Wing loading and habitat selection in forest beetles: Are red-listed species poorer dispersers or more habitat-specific than common congenerics? *Biol. Conserv*. **132**, 250–260 (2006).

27. Osborne, M. F. M. Aerodynamics of flapping flight with application to insects. *J. Exp. Biol*. **28**, 221–245 (1951).

28. Larsén, O. On the morphology and function of the locomotor organs of the Gyrinidae and other Coleoptera. *Opuscula Entomologica (Supplementum)* **30**, 1–241. (1966)

29. Ellington, C. P. Power and efficiency of insect flight muscle. *J. Exp. Biol*. **115**, (1985).
