# Supplementary material for: Novel flight style and light wings boost flight performance of tiny beetles
Source: Nature. 2022 Jan 19;602(7895):96–100. doi: 10.1038/s41586-021-04303-7 (PMC8810381; doi:10.1038/s41586-021-04303-7)
Supplement: Supplementary file 2 — Reporting Summary [file 41586_2021_4303_MOESM2_ESM.pdf]

nature research

Corresponding author(s): Alexey Polilov
Sergey Farisenkov

Last updated by author(s): Nov 4, 2021

Reporting Summary

Nature Research wishes to improve the reproducibility of the work that we publish. This form provides structure for consistency and transparency in reporting. For further information on Nature Research policies, see our Editorial Policies and the Editorial Policy Checklist.

Statistics

For all statistical analyses, confirm that the following items are present in the figure legend, table legend, main text, or Methods section.

n/a	Confirmed	
☐	☒	The exact sample size (*n*) for each experimental group/condition, given as a discrete number and unit of measurement
☐	☒	A statement on whether measurements were taken from distinct samples or whether the same sample was measured repeatedly
☐	☒	The statistical test(s) used AND whether they are one- or two-sided<br>*Only common tests should be described solely by name; describe more complex techniques in the Methods section.*
☐	☒	A description of all covariates tested
☐	☒	A description of any assumptions or corrections, such as tests of normality and adjustment for multiple comparisons
☐	☒	A full description of the statistical parameters including central tendency (e.g. means) or other basic estimates (e.g. regression coefficient) AND variation (e.g. standard deviation) or associated estimates of uncertainty (e.g. confidence intervals)
☐	☒	For null hypothesis testing, the test statistic (e.g. *F*, *t*, *r*) with confidence intervals, effect sizes, degrees of freedom and *P* value noted<br>*Give P values as exact values whenever suitable.*
☒	☐	For Bayesian analysis, information on the choice of priors and Markov chain Monte Carlo settings
☒	☐	For hierarchical and complex designs, identification of the appropriate level for tests and full reporting of outcomes
☒	☐	Estimates of effect sizes (e.g. Cohen's *d*, Pearson's *r*), indicating how they were calculated

Our web collection on statistics for biologists contains articles on many of the points above.

Software and code

Policy information about availability of computer code

Data collection	The high-speed recordings were filmed in Evercam SRV-HS. Microphotographs were received in Tucsen Mosaic 2.0. 3D reconstruction of body parts were performed in Bitplane Imaris v.9.5.<br>Non-standard or custom-designed programmes or codes were not used.
Data analysis	Primary video processing was performed using ImageJ v.1.52p. Morphometric analysis were performed using Autodesk AutoCAD 2015.Kinematic data processing were performed using Autodesk 3ds Max 15.0 and the standard packages of Matlab R2019b v.3.1 and open-source R packages in R v.4.0.0.28 enviroment. Computational fluid dynamics simulations were performed using an open-source code WABBIT. It can be downloaded from GitHub (https://github.com/adaptive-cfd/WABBIT) and it has been described in detail elsewhere [30].

For manuscripts utilizing custom algorithms or software that are central to the research but not yet described in published literature, software must be made available to editors and reviewers. We strongly encourage code deposition in a community repository (e.g. GitHub). See the Nature Research guidelines for submitting code & software for further information.

Data

Policy information about availability of data

All manuscripts must include a data availability statement. This statement should provide the following information, where applicable:
- Accession codes, unique identifiers, or web links for publicly available datasets
- A list of figures that have associated raw data
- A description of any restrictions on data availability

Extended data sets and faw data are available in the following Open Science Framework repository: https://osf.io/v3wrk/

Field-specific reporting

Please select the one below that is the best fit for your research. If you are not sure, read the appropriate sections before making your selection.

☒ Life sciences ☐ Behavioural & social sciences ☐ Ecological, evolutionary & environmental sciences

For a reference copy of the document with all sections, see nature.com/documents/nr-reporting-summary-flat.pdf

Life sciences study design

All studies must disclose on these points even when the disclosure is negative.

Sample size	Sample sizes for kinematic reconstruction and analysis, and CFD analysis were determined by quantity and quality of high-speed videos available. The complete reconstruction of the kinematics and CFD was done for four individual beetles, four wing beat cycles of each beetle. The other characteristics of the kinematics were measured using all the 13 recordings or some of them. Each recording contains 3 to 23 full wing beats.<br>The sample sizes for morphometrics comprised at least 10 replications. Statistical methods were not used to predetermine the sample size. Sample sizes were selected based on the limited amount of material available.
Data exclusions	No data were excluded.
Replication	Experiments were performed independently.
Randomization	Artificial randomization is not relevant to this study because the original video recordings were obtained from random samples, and then all of them were analysed.
Blinding	Blinding was not possible because the analysis of the data was carried out by the persons involved in the data collection, and the interpretation of the data was carried out by the persons responsible for the analysis.

Reporting for specific materials, systems and methods

We require information from authors about some types of materials, experimental systems and methods used in many studies. Here, indicate whether each material, system or method listed is relevant to your study. If you are not sure if a list item applies to your research, read the appropriate section before selecting a response.

Materials & experimental systems

n/a	Involved in the study	
☒	☐	Antibodies
☒	☐	Eukaryotic cell lines
☒	☐	Palaeontology and archaeology
☐	☒	Animals and other organisms
☒	☐	Human research participants
☒	☐	Clinical data
☒	☐	Dual use research of concern

Methods

n/a	Involved in the study	
☒	☐	ChIP-seq
☒	☐	Flow cytometry
☒	☐	MRI-based neuroimaging

Animals and other organisms

Policy information about studies involving animals; ARRIVE guidelines recommended for reporting animal research

Laboratory animals	The study did not involve laboratory animals.
Wild animals	The featherwing beetle Paratuposa placentis (Coleoptrea: Ptiliidae) is a widespread and abundant species. It is not a protected species, and working with it does not require special permits. The sex of the beetles was not determined, because this species has no pronounced sexual dimorphism, and the sex of an individual beetle cannot be determined without dissecting, which would have been incompatible with most of the methods used. The study was performed on adult beetles; their exact age could not be determined, because the beetles were taken from nature, and no characters that can be used to determine the age of adult beetles of this species are known.<br>The beetles were captured in the natural habitat with the substrate and brought to the laboratory in containers for recording their flight. After the video recording session, most beetles were released in the collection locality in the same day, and only the minimum required number of specimens for the morphological part of the study The insects were anesthetized with CO_2 and then fixed in Bouin's splution or 70% ethanol according to the standard method.
Field-collected samples	The beetles were kept in ventilated containers with the substrate for several hours after capture in laboratory (temperature 22-24 degree, natural lighting conditions) and were released or fixed after experiments.

Ethics oversight	Ethics oversight was not required because ptiliid beetles do not require IRB or ethics approval. The studied beetles do not belong to protected or endangered species.<br>Field work was carried out in the framework agreement № 37/HD on the scientific cooperation between Cat Tien National Park and the Joint Russian–Vietnamese Tropical Research and Technological Centre.

Note that full information on the approval of the study protocol must also be provided in the manuscript.
